# Individual Cortical Entropy Profile: Test–Retest Reliability, Predictive Power for Cognitive Ability, and Neuroanatomical Foundation

**DOI:** 10.1093/texcom/tgaa015

**Published:** 2020-05-07

**Authors:** Mianxin Liu, Xinyang Liu, Andrea Hildebrandt, Changsong Zhou

**Affiliations:** 1 Department of Physics, Centre for Nonlinear Studies and Beijing-Hong Kong-Singapore Joint Centre for Nonlinear and Complex Systems (Hong Kong), Institute of Computational and Theoretical Studies, Hong Kong Baptist University, Kowloon Tong, Hong Kong; 2 Department of Psychology, Carl von Ossietzky Universität Oldenburg, 26129 Oldenburg, Germany; 3 Department of Physics, Zhejiang University, 310000 Hangzhou, China

**Keywords:** brain signal entropy, cognitive ability prediction, individual differences, neuroanatomical basis, test–retest reliability

## Abstract

The entropy profiles of cortical activity have become novel perspectives to investigate individual differences in behavior. However, previous studies have neglected foundational aspects of individual entropy profiles, that is, the test–retest reliability, the predictive power for cognitive ability in out-of-sample data, and the underlying neuroanatomical basis. We explored these issues in a large young healthy adult dataset (Human Connectome Project, *N* = 998). We showed the whole cortical entropy profile from resting-state functional magnetic resonance imaging is a robust personalized measure, while subsystem profiles exhibited heterogeneous reliabilities. The limbic network exhibited lowest reliability. We tested the out-of-sample predictive power for general and specific cognitive abilities based on reliable cortical entropy profiles. The default mode and visual networks are most crucial when predicting general cognitive ability. We investigated the anatomical features underlying cross-region and cross-individual variations in cortical entropy profiles. Cortical thickness and structural connectivity explained spatial variations in the group-averaged entropy profile. Cortical folding and myelination in the attention and frontoparietal networks determined predominantly individual cortical entropy profile. This study lays foundations for brain-entropy-based studies on individual differences to understand cognitive ability and related pathologies. These findings broaden our understanding of the associations between neural structures, functional dynamics, and cognitive ability.

## Introduction

Spontaneous neural activity is often characterized by persistent and highly irregular fluctuations. According to the prevalent theoretical view, this irregularity can be explained by a balance between excitatory and inhibitory interneuron currents, which is underpinned by local neural circuits and networks (Van Vreeswijk and Sompolinsky 1996; Shu et al. 2003). Studies on brain systems have associated the variability or complexity of ongoing neural fluctuations with the information capacity (Shew et al. 2011), the dynamic range of responses to stimuli (Shew et al. 2009), and the observed flexible phase synchrony (Yang et al. 2012) of brain systems. The generality of the underlying neurological mechanisms and functional implications suggests that the complexity of spatiotemporal patterns in brain activity serves as suitable biomarkers of brain functions and disorders (Faisal et al. 2008; McIntosh et al. 2008, 2010; Garrett et al. 2010, 2011, 2013a, 2013b, 2014; Takahashi 2013; Guitart-Masip et al. 2016).

Studies on cognitive neuroscience have widely applied entropy as a representative and quantitative measure of complexity in brain dynamics. When resting-state signals captured by electroencephalography (EEG) or functional magnetic resonance imaging (fMRI) were investigated, group comparisons with respect to the cortical entropy *profile* (spatial complex pattern) were applied to reveal substantial differences between typically and atypically functioning populations, such as people under development (McIntosh et al. 2008, 2010; Lippé et al. 2009; Mišić et al. 2010; Vakorin et al. 2011), aging (Takahashi et al. 2009; O’Hora et al. 2013; McIntosh et al. 2014; Jia et al. 2017), traumatic brain injury (Raja Beharelle et al. 2012), schizophrenia (Takahashi et al. 2010; Xue et al. 2019), depression (Okazaki et al. 2013; Lin et al. 2019), autism (Bosl et al. 2011; Okazaki et al. 2015; Takahashi et al. 2016; Liu et al. 2017; Bosl et al. 2018; Easson and McIntosh 2019; Kang et al. 2019) and Alzheimer’s disease (Mizuno et al. 2010; Yang et al. 2013; Azami et al. 2017; Wang et al. 2017).

Recently, the investigation of brain-wide activity in terms of individual deviations from the group average has become a crucial endeavor in the context of individualized predictions and precision medicine. Compared with group contrast, individual differences within group are more difficult to detect but more essential to the characterization of brain function in health and disease (Dubois and Adolphs 2016). Majority of researchers used functional connectivity (FC) and have attempted to examine individual brain functions related to healthy aging (Dosenbach et al. 2010; Geerligset al. 2015), personality (Adelstein et al. 2011; Dubois et al. 2018a), intelligence (Finn et al. 2015; Dubois et al. 2018b), and disease (Arbabshirani et al. 2013; Yahata et al. 2016; Easson et al. 2019). The application of entropy measure in the field of individual difference is just emerging. Early work recorded resting-state EEG from healthy elderly subjects and found higher individual creativity was linked to the increased entropy especially in lower frequencies (Ueno et al. 2015). A very recent study in young healthy adults further explored the association between the voxel-level-resolution whole-brain resting-state fMRI (rfMRI) entropy profiles and individual differences in creativity (Shi et al. 2019). Consistently, the study revealed that high entropy in the control and semantic associative networks was associated with superior creative ability across individuals. Another work in rfMRI of young healthy subjects observed positive correlation between individual scores from vocabulary and reasoning tasks and brain entropy in prefrontal cortex, inferior temporal lobes, and cerebellum (Saxe et al. 2018). These studies gave first evidences about the sensitivity of entropy profile as novel marker to capture individual difference in cognitive processing.

However, several crucial aspects should be elucidated before further exploration of individual difference using entropy profile. First, the existing literature (Zuo and Xing 2014; Zuo et al. 2014, 2019a; Dubois and Adolphs 2016; Xing and Zuo 2018; Zuo et al., 2019b) emphasizes the status of reliability (i.e., reproducibility in test–retest trials) and validity (i.e., the accurate measurement of functionally relevant information) as foundations of the brain science of individual differences. The low reliability is known to potentially cause false positives, false negatives, and/or artificially inflated effect sizes (Zuo et al., 2019a). In addition, the restricted reliability could bias the evaluation of functional significance of different brain subsystems, since the reliability sets the upper bound of measurements’ validity (Zuo et al., 2019b). Previous studies on the entropy-based measures of whole-brain dynamics have lacked these aspects, and it is not known if the reliability of entropy in different subsystems is heterogeneous. Furthermore, an out-of-sample prediction should be favored over a correlation analysis, as the latter derives conclusions based on inferences from the in-sample population and does not directly test generalizability (predictive validity) of entropy measure in out-of-sample population (Linden 2012; Whelan and Garavan 2014; Gabrieli et al. 2015; Lo et al. 2015). As with group average comparisons, simple correlation-based analyses are performed separately for spatial sites. Thus, previous research did not consider the cortical entropy profile as an integrative measure of dynamic activity in the brain and did not include out-of-sample predictions of cognitive ability. Moreover, an understanding of the anatomical basis underlying spatial heterogeneity and individual differences in cortical entropy profiles remains lacking, despite the critical nature of this information to the future development of clinical diagnosis and treatment.

In summary, studies on entropy-based individual differences must urgently address 3 fundamental questions: (1) Are the individual cortical entropy profiles in the whole cortex and functional subsystems reliable and person-specific trait indicators? (2) When the profile is reliable, is the cortical entropy profile predictive of the cognitive ability of an out-of-sample individual? (3) Which anatomical features determine spatial heterogeneity and individual differences in cortical entropy profiles?

In this study, we used a large dataset from the Human Connectome Project (HCP) (Van Essen et al. 2013) to address these questions. First, we aimed to investigate whether individual profiles of cortical entropy can be measured reliably and are sufficiently unique to serve as a reliable individual “fingerprint.” Second, we aimed to use integrated information from whole-cortex entropy profiles in a predictive modelling framework to predict individual general and specific cognitive abilities. Third, we aimed to quantify the anatomical basis of the spatial heterogeneity observed in group-averaged entropy profiles. Finally, we searched for the anatomical features that influence the prediction of individual entropy profiles.

## Materials and Methods

### The HCP Database

We examined the data of 998 healthy young adults (age range: 22–35 years), including 466 males, from the HCP S1200 data release (Van Essen et al. 2013). Participants with missing data from any imaging modality were excluded. The HCP provides data for public use, including scans obtained using different MRI modalities in a large population. The database includes property maps from structural MRI [T1-weighted (T1w) and T2-weighted (T2w) imaging], diffusion MRI (dMRI), rfMRI, and outside scanner cognitive performance data. Detailed protocols used to acquire MRI data for the HCP are provided at https://www.humanconnectome.org and have been described extensively in the literature (Van Essen et al. 2013).

#### Structural MRI

Structural MRI data comprises T1w and T2w images. T1w images were obtained using the T1w_MPR1 sequence with the following parameters: repetition time (TR) = 2400 ms, echo time (TE) = 2.14 ms, flip angle = 8, field of view = 224 × 224 (RO × PE), and isotropic voxels = 0.7 mm. T2w images were obtained using the T2w_SPC1 sequence with the following parameters: TR = 3200 ms, TE = 565 ms, field of view = 224 × 224 (RO × PE), and voxel size = 0.7 mm. All structural data were preprocessed through the HCP minimal preprocessing pipeline (Glasser et al. 2013), specifically, surface-based “CIFTI” format data, which comprised 91 282 cortical and subcortical “grayordinates” (Glasser et al. 2013).

Surface-based morphometry measurements, including the cortical thickness, mean cortical curvature (curv), and sulcus depth (sulc), were determined based on the structural MRI data. Thickness was defined as the distance between the gray and white matter border and the pial surface. Curv represented the curvature of each vertex on the cortical surface. A higher curv value indicated sharper bending, and a positive value corresponded to curvatures in sulci (i.e., upward curving). Sulc was defined as the linear distance from the midsurface between the gyri (negative value) and sulci (positive value). A previous empirical study determined that the ratio of T1w to T2w (T1/T2) could be considered as a proxy of the myelin content (Glasser and Van Essen 2011). The HCP dataset provides these measures at both individual and group levels and can be downloaded directly from the official HCP website.

#### Diffusion MRI

The dMRI data were acquired using a spin-echo EPI sequence with the following parameters: TR = 5520 ms, TE = 89.5 ms, flip angle = 78, field of view = 210 × 180 (RO × PE), matrix = 168 × 144 (RO × PE), slice thickness = 1.25 mm, slice number = 111, isotropic voxels = 1.25 mm, echo spacing = 0.78 ms, BW = 1488 Hz/Px and *b*-values = 1000, 2000, and 3000 s/mm^2^. The sampled data were preprocessed using the minimal preprocessing pipeline (Glasser et al. 2013).

#### Resting-State fMRI

Four rfMRI scans were sampled, and each run duration was approximately 15 min. The runs were recorded over two sessions on different days (REST1 and REST2). Each session included a right-to-left (RL) scan during 1 run and a left-to-right (LR) scan during the other run. The following scanner parameters were used: gradient-echo EPI sequence, TR = 720 ms, TE = 33.1 ms, flip angle = 52, field of view = 208 × 180 (RO × PE), matrix = 104 × 90 (RO × PE), slice thickness = 2 mm, slice number = 72, isotropic voxels = 2.0 mm, multiband factor = 8, echo spacing = 0.58 ms, and bandwidth = 2290 Hz/Px.

As with the other imaging modalities, the HCP provides preprocessed rfMRI data that have been subjected to a minimal preprocessing pipeline (Glasser et al. 2013). The data were denoised further using the ICA (independent component analysis)-based Xnoiseifier (FIX) (Griffanti et al. 2014; Salimi-Khorshidi et al. 2014). CIFTI format data were used to match the structural MRI data with the rfMRI data. The signals were further filtered through a 0.01-Hz high-pass filter to remove slow drifts induced by the scanner.

### Brain Parcellation

**Figure 1:**
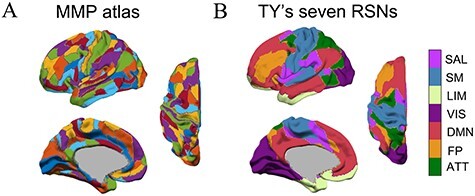
Depiction of the MMP atlas and mapping of MMP regions to Thomas Yeo’s 7 RSNs. (*A*) An MMP atlas of the left hemisphere is displayed. (*B*) The MMP atlas is overlaid on the corresponding 7 functional networks from Thomas Yeo et al.’s atlas. SAL, salience network (49 ROIs in 2 hemispheres); SM, somatomotor network (52 ROIs); LIM, limbic network (28 ROIs); VIS: visual network (59 ROIs); DMN: default mode network (83 ROIs); FP, frontoparietal network (45 ROIs); ATT, attention network (44 ROIs). According to the cortical hierarchy hypothesis, the SM and VIS networks are lower-order sensory systems, whereas the other networks are higher-order association systems (Huntenburg et al. 2018).

We combined two brain parcellation atlases for our analyses. The Multi-modal Parcellation (MMP) atlas (Glasser et al. 2016) defines 180 brain areas in each hemisphere. These areas can be used as seed regions to extract fMRI time series and structural properties. To describe the functional specificity, these 180 MMP regions were further matched to a classical brain functional parcellation based on the resting-state network (RSN) (Thomas Yeo et al. 2011). This analysis considered the parcellation of 7 RSNs. We assigned each MMP region to the corresponding region among the 7 functional networks in Thomas Yeo et al.’s (2011) atlas by identifying the highest overlapping rate between the atlases. The regional matches between the two atlases are depicted in Figure 1.

### Brain Structural Connectivity

For each individual, fiber tractography was used to construct a 360 × 360 structural connectivity matrix based on the dMRI data. First, the Connectome Workbench (Van Essen et al. 2013) was used to back project all MMP areas defined in the standard space to individual cortical surfaces in the diffusion space. FSL software (version 5.0.9, Behrens et al. 2003, 2007) was then used to perform probabilistic tractography between every pair of ROIs. During pair-wise fiber tracing, 1 region was used as the seed area and the other as the target and vice versa. A total of 5000 streamlines were sent from each vertex of the seed ROI. Two thousand steps of streamline propagation were performed with a step length of 0.5 mm and a curvature threshold of 0.2. Individual fractional anisotropy (FA) maps based on preprocessed dMRI data were generated using FSL. Fiber streamline propagation would cease when a voxel with an FA value of <0.1 or the brain pial surface was encountered. The directional connective probability ${p}_{ij}$ from a seed ROI *i* to a target ROI *j* was calculated by dividing the number of streamlines that reached the target ROI by the total number of streamlines initiated from the seed ROI (5000 multiplied by the number of vertices in the seed ROI) (Behrens et al. 2007). A higher probability indicated stronger structural connectivity between the ROIs. Based on the calculated directional connective probability, we further defined the structural network weight ${w}_{ij}$, by calculating the reciprocal averages of the connective probabilities (i.e., ${w}_{ij}$ = (${p}_{ij}$+ ${p}_{ji}$)/2) (Cao et al. 2013). Next, the matrix was thresholded using values maintained above ${w}_{ij}$ = 0.001, which led to the retention of approximately 10% of the links in the individual structural networks (Cao et al. 2013) (see Supplementary Fig S1*A*). Additional details about the influence of thresholding are illustrated in Supplementary Figure S1.

Based on the structural connectivity matrices, we investigated two network measures being indicative of node centrality: (1) The connectivity strength *S_i_* was defined by summing all link weights (${w}_{ij}$) from 1 node to all others: ${S}_i={\sum}_j{w}_{ij}$. However, this calculation did not sufficiently reflect the contributions of links with relatively small ${w}_{ij}$ values (i.e., weak links). (2) The connectivity degree *K_i_* was equal to the number of existing links to 1 node after thresholding. This definition balanced the effects of weak and strong links above the threshold.

### Complexity of Neuronal Activity

In our previous work (Liu et al. 2019), we used a measure called “dispersion entropy” to characterize the complexity of neuronal signals (Azami et al. 2017; Rostaghi and Azami 2016). Compared with other measures of complexity, dispersion entropy was relatively robust under the presence of noise in the analyzed time series and yielded a high computation efficiency (*O*(*N*)). Besides, when applied to neuron time series data, dispersion entropy and sample entropy yielded similar results (Kuntzelman et al., 2018). And the single scale dispersion entropy can more reliably assess the underlying neuronal spatiotemporal variability than the multiscale dispersion entropy at high temporal scales (Liu et al. 2019).

Dispersion entropy (Azami et al. 2017; Rostaghi and Azami 2016) aims to reveal the dispersion patterns (i.e., symbolic dynamics) embedded in the signal and to use Shannon entropy to quantify the complexity of the appearances of these patterns. The input signal is denoted as $S$. First, when referring to the normal cumulative distribution function (NCDF), the value at every time point in the fMRI signal is converted to the class variable label ${z}_i$ (*c* classes, and thus ${z}_i$ values, range from 1 to *c*) through 2 steps of mapping:

$$ {\theta}_i=1/D\sqrt{2\pi }{\int}_{-\infty}^{s_i}\exp \left(-\left(s-M\right)/2{D}^2\right) ds, $$

followed by:

$$ {z}_i=\mathrm{round}\left(c\ast{\theta}_i+0.5\right). $$

The mean *M* and standard derivation *D* of the NCDF are given by the mean and the standard deviation of the signal$S$, respectively. Next, a sliding window of length *m* (i.e., embedding dimension) is used to bin the sequence $z$ into dispersion patterns [${z}_i$,.., ${z}_{i+m}$], and the step length of the sliding window is defined by $\tau$ (i.e., time delay). After the window has scanned the whole signal, the probability *p* of each detected dispersion pattern is used to calculate the entropy:

$$ \mathrm{EN}=-\sum_i{p}_i\log \big({p}_i\big). $$

Theoretically, the upper bound of dispersion entropy could be defined as the point where the signal behaves as completely random noise and all dispersion patterns can coexist with identical probability. The lower bound could be achieved if the signal is periodic. While the choice of the parameters *c*, *τ*, and *m* will differently scale the entropy estimates for a given time series, we found that parameter setting will not alter the estimation of the individual entropy profile (see Supplementary Fig. S2, the rank order of entropy estimates across ROIs remained same). For all subsequent analyses, we fixed the parameters to the following values: *c* = 3, *τ* = 1, and *m* = 2.

The codes for computing dispersion entropy can be found in https://datashare.is.ed.ac.uk/handle/10283/2637.

### Fingerprint Identification

**Figure 2:**
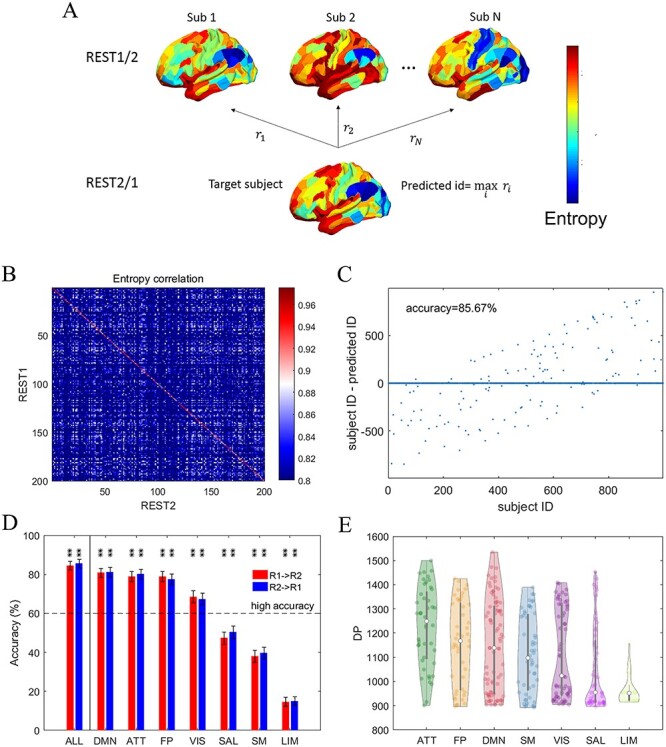
The individual cortical entropy profile as a fingerprint. (*A*) Depiction of the identification procedure. We used the entropy profile measured on 1 day (REST1) to compute the correlations between this profile and all other individuals’ profiles generated by measurements on the other day (REST2). The predicted ID was defined as the participant’s ID that yielded the highest correlation coefficient. (*B*) The correlation matrix of a group of 200 randomly selected participants used to illustrate fingerprint identification based on whole-cortex entropy profiles. (*C*) A scatter plot of the enquired participant IDs versus the difference between enquired and predicted IDs using REST2 (database set) and REST1 data (target set), namely “R2 → R1”, based on whole-cortex entropy profiles. (*D*) A bar plot of accuracy when REST1 was used to predict REST2 and vice versa. Entropy profiles from the whole cortex (ALL), and each of the 7 RSNs was used to compute identification accuracy. The RSNs are ranked by the accuracy of “R1 → R2”. ^*^ and ^**^ above the bars denote the corresponding significance levels of *P* < 0.05 and *P* < 0.01 obtained by the permutation test, respectively, after applying the correction of FDR < 0.05. The error bars indicate the upper and lower boundary of the 95% CI provided by bootstrapping. (*E*) A violin plot of the distribution of differentiation power (DP) across cortical areas in each of the 7 RSNs. The RSNs are ranked by the DP distribution median values.

“Fingerprint” identification (Finn et al. 2015) was first performed to prove the existence of substantial individual differences in cortical entropy profiles (Fig. 2*A*). Here, we denoted the vector of the individual entropy profile of participant *i* on day *d* as ${\overrightarrow{\mathrm{EN}}}_i^d$, where $1\le i\le N$ and *d =* 1 or 2. Next, we defined an identification procedure. Of the 2-day measurements, the entropy profile from 1 day was assigned as the “target set” for which the participant ID was supposed to remain unknown. The profile measured on a different day was selected as the “database set.” The identification process aimed to predict the ID of a participant in the target group based on information from the database set. The predicted ID of the target was obtained by searching the database set for the participant with the most similar entropy profile characteristics. Formally, by setting the input entropy profile as ${\overrightarrow{\mathrm{EN}}}_i^{d1}$ and the data in database set as ${\overrightarrow{\mathrm{EN}}}_j^{d2}$, we computed the correlation as:

$$ r\left(i,j\right)=\mathrm{corr}\left({\overrightarrow{\mathrm{EN}}}_i^{d1},{\overrightarrow{\mathrm{EN}}}_j^{d2}\right),{d}_1\ne{d}_2, $$

where *i* is the enquired participant and *j* is the database set participant. The predicted ID is defined for *j* with the maximal similarity to *i* in the database set, namely ${\max}_{j}\,r\big(i,j\big).$As Finn et al. (2015), we used a nonparametric approach to assess the significance of the identification accuracy, more specifically with a permutation test. After obtaining the predicted ID based on profile matching, the participant ID in the target set was permuted such that the original prediction was compared with a set of not-paired observations. We first randomly chose data from 1 of the measurement days as the database and the other day as the target set, and conducted permutations 500 times. The roles of the datasets were then reversed, and another 500 permutations were performed. The final *P*-value was computed as the probability of achieving an accurate result that was greater than or equal to the original accuracy over the 1000 total permutations. However, we observed a maximum accuracy of only 0.60% across all realizations of the permutation test (only 6 participants matched; please refer to Supplementary Fig. S3 for an example), which is not acceptable. Thus, significance does not guarantee a high accuracy. According to the psychometric literature on reliability (Coaley 2014), we applied a tighter criterion besides significance, namely that the accuracy needs to be larger than 60% to be considered satisfactory. If the identification was successful (i.e., accuracy was above 60%), we concluded that the entropy profiles were stable within individuals, but varied across individuals, and could thus be considered an individual “fingerprint.”

### Differentiation Power

Different ROIs may contribute differently to the identification process. Finn et al. (2015) proposed a metric called differentiation power (DP) to quantify the elementary contributors to identification. The Pearson correlation coefficient is the sum of the element-wise products of 2 *z*-scored vectors. Therefore, given ${\overrightarrow{z\mathrm{EN}}}_i^{d1},\mathrm{z}{\overrightarrow{\mathrm{EN}}}_i^{d2}$ after normalization, the element-wise products could be represented as

$$ {\varphi}_{ij}(n)={\mathrm{zEN}}_i^{d1}(n)\ast{\mathrm{zEN}}_j^{d2}(n),n=1,2,..,360 $$

where ${\mathrm{zEN}}_i^d(n)$ represents the entropy value in the *n*th ROI of participant *i* on day *d* after *z*-score normalization and the sum $\sum_e{\varphi}_{ij}(n)$ represents the Pearson correlation between ${\overrightarrow{\mathrm{EN}}}_i^{d1}$and ${\overrightarrow{\mathrm{EN}}}_j^{d2}$. An effective identification requires the intraparticipant correlation be larger than the interparticipant correlation. Therefore, for ROIs that contribute to identification, the following property must remain true:

$$ {\varphi}_{ii}(n)>{\varphi}_{ij}(n)\ \mathrm{or}\ {\varphi}_{ii}(n)>{\varphi}_{ji}(n),i\ne j. $$

Under this condition, a given ROI contributes to the difference between the intraparticipant correlation and the cross-participant correlation. We can then compute the empirical probability as

$$\begin{eqnarray*} &&{P}_i(e)=P\left({\varphi}_{ij}(n)>{\varphi}_{ii}(n)\ \mathrm{or}\ {\varphi}_{ji}(n)>{\varphi}_{ii}(n)\right)= \\ && \qquad \frac{\left(\left|{\varphi}_{ij}(n)>{\varphi}_{ii}(n)\right|+\left|{\varphi}_{ji}(n)>{\varphi}_{ii}(n)\right|\right)}{2\left(N-1\right)},N=998. \end{eqnarray*}$$

A smaller ${P}_i(n)$ enhances the identification power of the ROI $n$ for participant *i* and can be interpreted similarly as the *P* value in standard statistical testing. The overall DP for the ROI $n$ can be obtained by averaging the ${P}_i(n)$ across individuals and calculating its negative logarithm:

$$ \mathrm{DP}(n)=-\ln \left(\sum_i{P}_i(n)\right). $$

A high or low DP value suggests that a given ROI has a greater or lesser contribution to identification, respectively.

### Regional Test–Retest Reliability

Test–retest reliability for each ROI was tested via the correlation of entropy at the same ROI between REST1 and REST2 across the 998 participants.

### Empirical Entropy Distribution of Random Signals

To identify noise contaminated ROIs, we constructed the empirical entropy distribution of random signals. The signals from each ROI of each subject in the first scan were temporally permutated. Then, entropy was calculated from such randomized time series for all the ROIs and all subjects, and these entropy values were investigated with respect to their deviation, forming the empirical entropy distribution of random signals. Entropy of the real data can thus be compared with this distribution to assess if the measured signal is distinguishable from random signals.

### Estimation of Cognitive Ability Scores

A comprehensive psychometric test battery that covered several dimensions of cognitive ability was applied to the HCP sample (Van Essen et al. 2013). Previous studies have used factor analyses to demonstrate that the performance scores generated by the measures included in the battery yield a reliable estimate of general cognitive ability (e.g., Dubois et al. 2018b). In this study, we adopted the established cognitive ability structure model proposed by Dubois et al. (2018b) to derive ability scores for use in subsequent predictive modelling. The model estimated a general cognitive ability factor that included the loadings from all task scores and 4 nested factors representing specific abilities (Fig. 4*A*). To better identify each cognitive task indicator in the model, we used the original abbreviated task names released by the HCP dataset (see Barch et al. 2013 for task descriptions). The specific factors were (1) *vis* (visuospatial ability), indicated by PMAT24_A_CR and VSPLOT_TC; (2) *cry* (crystallized intelligence), which included PicVocab_Unadj and ReadEng_Unadj; (3) *mem* (memory), indicated by IWRD_TOT, PicSeq_Unadj, and ListSort_Unadjfactor; and (4) *spd* (processing speed), indicated by CardSort_Unadj, Flanker_Unadj, and ProcSpeed_Unadj. The model fitted using confirmatory factor analysis (CFA) in the *lavaan* package (version 0.6-5, Rosseel 2012) for the R Software for Statistical Computing platform (version 3.6.1, R Development Core Team 2011). This nested factor model requires orthogonality among all factors. Missing behavioral task data were addressed using the full information maximum likelihood method as implemented in lavaan. Model fit was quantified using the ${\chi}^2$-goodness of fit test, the comparative fit index (CFI), the root mean squared error of approximation (RMSEA), and the standardized root mean square residual (SRMR). The CFI value of an acceptable model should exceed 0.95, whereas both the SRMR and RMSEA values should be <0.08 (Hu and Bentler 1999).

After constructing a well-fitting confirmatory factor model, we derived individual factor scores from the latent space for subsequent predictive modelling. Factor scores are indeterminate and therefore can be estimated from the latent space using different methods (Grice 2001). We applied the “Thurstone” regression-based method (using the *lavPredict* function in lavaan) and validated this estimation by evaluating the correlations (1) between the factors and the generated factor scores and (2) between the generated factor scores themselves. The analysis revealed strong correlations between the factors and their derived scores (see Supplementary Fig. S4*A*), suggesting that the scores were sufficiently consistent with the factors in terms of interindividual variations. However, the factor scores were not completely orthogonal to each other as required by the CFA model (see Supplementary Fig. S4*B*), because of indeterminacy.

### Prediction of Cognitive Ability Scores by Cortical Entropy Profiles

We performed a multivariate linear regression to predict the cognitive performance scores of participants based on the estimated individual differences in cortical entropy profiles.

We first trained a linear model based on the individual entropy profile:

$$ Y= bX+k, $$

where $Y$ is the column vector containing all individual factor scores and $X$ represents a matrix of individual entropy profiles in which the columns represent the ROIs. All columns were *z*-scored. To accommodate the large number of predictors and potential multicollinearity between these predictors, we used ridge regression regularization to estimate the model coefficients *b* and *k*. Mathematically, potential multicollinearity could lead to ill-conditioned covariance matrix when estimating *b* using the following equation:

$$ \overset{\sim }{b}={\left({X}^TX\right)}^{-1}\left({X}^TY\right). $$

The ridge regression is an extension of the ordinary least square regression, which minimizes both the deviation between the observed and predicted values (i.e., error) and the regularization term:

$$ \underset{b,k}{\min}\sum_i{\left({b}_i{x}_i-{y}_i\right)}^2+\lambda \sum_i{\left({b}_i\right)}^2. $$

Consequently, the ill-condition problem can be relieved because the estimation is revised as

$$ \overset{\sim }{b}={\left({X}^TX+\lambda I\right)}^{-1}\left({X}^TY\right), $$

where $I$ is the identity matrix.

Leave-one-family-out cross-validation (LOFOV) was used to avoid coupling between the training and test sets due to the family structure of the HCP sample (Dubois et al. 2018b). Literally, participants belonging to the same family were selected as the test dataset, and a model was constructed for participants belonging to other families. Each family was left out once throughout the iterations. After yielding estimations for all subjects, we assessed the accuracy of the predictions by correlating the observed and predicted scores of all participants. A negative correlation between the observed and predicted values might present due to noise or bad fitting and was thus fixed at zero. The results are provided for the ridge coefficient $\lambda$ that maximized the correlation in the LOFOV.

In previous studies based on FC profile, a feature selection phase was applied prior to training the multivariate model. In this phase, correlations between predictors (e.g., entropy in each ROI) and cognitive performance score across participants were calculated in the training set. Subsequently, a significance threshold, ${p}_{\mathrm{th}}$, was used to select informative predictors. However, we determined that feature selection would have nonlinear effects on the predictive performance (see Supplementary Fig. S5; accuracy may either increase or decrease after feature selection). Therefore, we omitted the feature selection from our analysis.

### Prediction of Entropy Profiles Based on Individual Structural Profiles

To investigate the associations between the brain structure and complexity, we constructed an additional predictive model based on the interindividual variability in structural property profiles (Fig. 6*D*). For each ROI, an ordinary least-square regression was used to train a linear regression model to map the individual regional structural properties to the corresponding regional entropy value (REST1 and REST2 averaged). Furthermore, the predicted entropy values of individual subjects in each ROI were listed together to generate a final estimated prediction of the individual entropy profile based on structural properties. The above-described LOFOV approach was also applied to this prediction.

We assessed the accuracies of prediction for observed entropy profile from 2 aspects. First, we computed the correlation similarities between the paired prediction and observation in the same participant. Second, we quantified the specificity of the prediction by subjecting all participants to fingerprint identification. Here, the predicted entropy profile was considered the database set, and correlation similarities were used to inquire the observed entropy profiles. A high level of identification accuracy (>60%) would suggest that participant’s structural properties could predict the participant’s own entropy profile to a much better degree than the structural properties of any other individual. The corresponding *P* value was yielded by 1000 times permutation on the predicted ID to randomize the paired relationship between observations and predictions. This specificity would indicate that an individual’s entropy profile resulted from individual variations in structural profiles.

### Statistical Analysis

Pearson correlation analyses were performed using the *corr* function in the MATLAB software (MATLAB 2016a), which simultaneously generated the correlation and *P* value. The *P* value is computed by transforming the correlation to create a *t*-statistic, having degrees of freedom equal to the number of observations minus 2. The 95% confidence interval (CI) was obtained by 1000 bootstrap samples for correlation and identification accuracy. In cases of multiple testing, we used the Benjamini–Hochberg method to control the false discovery rate (FDR) by correcting the *P* values at an FDR of <0.05.

## Results

### The Test–Retest Reliability of Individual Cortical Entropy Profiles

We demonstrated the crucial influence of test–retest reliability on detecting individual differences in entropy based studies with fingerprint identification (Finn et al. 2015) applied to whole-cortex entropy profiles (Fig. 2*A*). Briefly, we correlated the individual entropy profile of 1 participant, *i*, sampled on 1 day with those of all participants *j* (*j* = 1–998) sampled on the other day. Only if the entropy profile was considered reliable within the participant and exclusive between participants, this correlation can peak at *j = i* and the identification can success. In the depicted correlation matrix (only 200 participants included for a clear illustration), most of the pronounced correlation values lie on the diagonal line *i = j* (Fig. 2*B*). When the participant ID *j* at the peak of each row was used as the predicted participant ID, we found that the predictions based on REST1 matched the targets from REST2 with accuracy levels as high as 85.67% [855 of 998 participants matched in the whole dataset (Fig. 2*C*)], *P* = 0.000, 95% CI = [83.47%, 87.88%], which fits our criteria of successful identification (accuracy > 60.00%, described in Materials and Methods). These results suggest that the whole-cortex entropy profile can be considered a reliable fingerprint. Note, however, that the achieved accuracy was slightly lower than that of FC profile-based identification in previous studies (Finn et al., 2015), which was applied to the same dataset (see Supplementary Fig. S6*A*).

However, we found the identification accuracy dropped when the scope was narrowed to specific functional systems. We repeated the identification procedure using the entropy profiles within each of the 7 RSNs (Fig. 2*D*). DMN, ATT, FP, and VIS succeeded the identification when considering 60% as a criterion. Notably, 3 networks, DMN, ATT, and FP, yielded the highest predictive accuracies, and these levels were only slightly lower than the results obtained from the whole-cortex analyses. The LIM network yielded the lowest levels of accuracy (Fig. 2*D*). These observations are consistent with our analysis of DP in ROI level (Fig. 2*E*), which assesses the contributions of different functional systems to the identification. The cortical DP distribution (see brain map in Supplementary Fig. S7*A*) was sorted into RSNs and is displayed as a violin plot in Figure 2*E*. The ROIs belonging to the ATT, FP, and DMN networks exhibited a high DP, and again LIM ranked in the last place.

**Figure 3:**
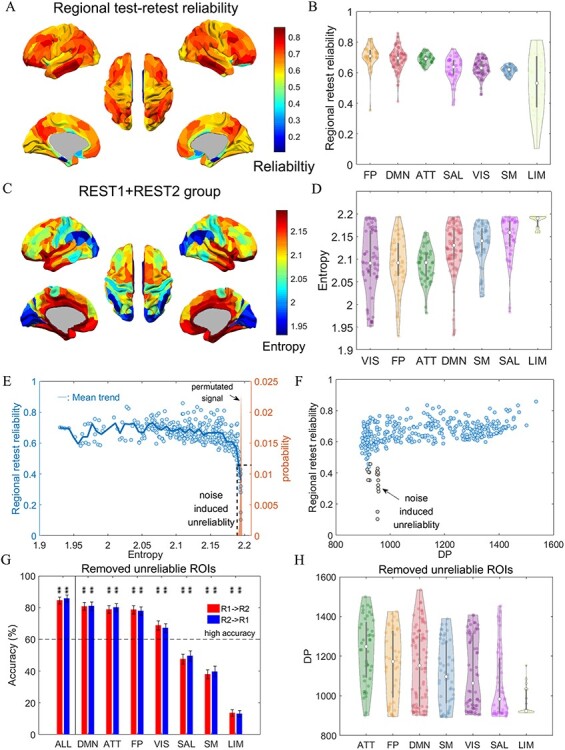
The noise induced unreliability in entropy profiles. (*A*) Brain map of regional test–retest reliability. (*B*) Violin plots for distribution of retest reliability in the 7 RSNs. (*C*) Brain map of group entropy profile averaged over all data (2 days and all participants). (*D*) Violin plots for distribution of group averaged entropy profile in the 7 RSNs. (*E*) Scatter plot of regional retest reliability against the regional group averaged entropy. The narrow distribution of entropy obtained from permutated signal of the first scan was given for comparison. (*F*) Scatter plot of regional retest reliability against DP. The unreliable ROIs identified in (*E*) are colored as gray. (*G*) The recomputed identification accuracy using entropy profile from whole cortical (ALL) and each RSNs after the removal of the unreliable ROIs. The legends are the same as Figure 2D. (*H*) The recomputed DP distribution of each RSNs after the unreliable ROIs is removed. The legends are the same as Figure 2E.

The heterogeneity with respect to identification accuracy and DP might have originated from either spatially heterogeneous individual differences or measurement precision (reliability). We computed and visualized the regional test–retest reliability in form of brain maps (Fig 3*A*) and violin plots representing different functional systems (Fig. 3*B*). The low reliability regions appear between the hemispheres, most of which belong to the LIM network. This suggests the low identification accuracy and low DP in LIM are a consequence of low retest reliability. This is to say that individual differences in neuronal activity cannot be properly captured in these regions with noisy signal. In addition, we plotted the whole-data averaged entropy profile (Fig. 3*C*) since the entropy measure is also sensitive to identify low signal-to-noise ratio. The figure illustrates that entropy in ROIs located between hemispheres appears higher as compared with other ROIs. When categorizing the ROIs into functional networks (Fig. 3*D*), the LIM network exhibits the highest median entropy, and most entropy estimates were at ceiling, similarly to random noise. Combining the 2 dimensions, we depicted the regional reliability against the regional entropy (Fig. 3*E*). The figure illustrates how the mean reliability starts to drop with increasing entropy, when entropy approaches to its maximum given by temporally permutated random signal. We conservatively evaluate noisy and thus unreliable ROIs as those regions with reliability below ${\mathrm{th}}_{\mathrm{relia}}=$ 0.4574 and entropy larger than ${\mathrm{th}}_{\mathrm{en}}=$ 2.1906. The 2 thresholds, ${\mathrm{th}}_{\mathrm{relia}}$ and ${\mathrm{th}}_{\mathrm{en}}$, are respectively the fifth quantile from the empirical reliability distribution and the permuted signal entropy distribution. As illustrated in Figure 3*F*, the identified ROIs were observed to have limited DP. This plot is in line with our understanding according to which part of the observed low DP, such as in LIM in Figure 2*F*, is mainly caused by noise-induced unreliability.

Since the validity of the ROIs is fundamentally restricted by their reliability, we excluded the detected ROIs with the lowest reliability/highest entropy from the following analyses. We thus compare the validity of subsystems with similar and satisfactory reliability only. This exclusion removed a total of 17 ROIs, including 1 ROI in FP, 1 ROI in DMN, 1 ROI in VIS, 11 ROIs in LIM, and 3 ROIs in SAL. After eliminating unreliable ROIs, we recalculated fingerprint identification accuracy and DP distribution (Fig. 3*G*,*H*), which are used for inference about the degree of individual differences in each RSN. This rather conservative data exclusion did not remarkably change the rankings among RSNs, compared to Figure 2*E*,*F*. The entropy profiles from DMN, ATT, and FP networks turned out to be the RSNs with the highest degree of individual differences. However, the retained number of ROIs belonging to LIM was very low (Fig. 3*H*). Note that using an even more conservative thresholding could further improve reliability while reducing the numbers of included ROIs in LIM, SAL, and VIS according to Figure 3*B*.

### Individual Cortical Entropy Profile as a Predictor of Cognitive Ability

**Figure 4:**
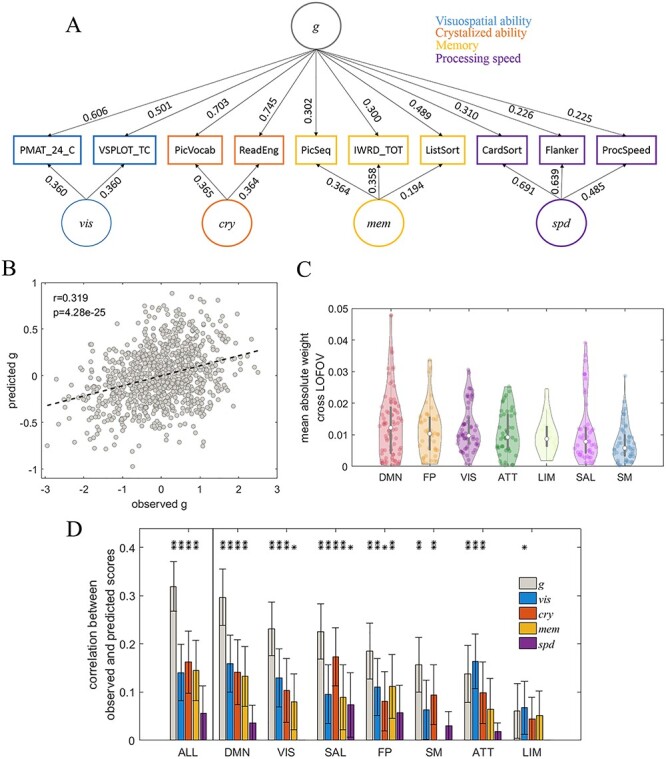
Prediction of the facets of cognitive ability using individual cortical entropy profiles. (*A*) The CFA model used to estimate cognitive ability scores, along with standardized factor loading estimates. (*B*) A scatter plot of the observed general cognitive ability factor scores *g* vs. predicted *g*, based on entropy profiles across the whole cortex. (*C*) Violin plots of the distributions of averaged absolute regression weights achieved across the LOFOV for each reliable ROI in a given RSN. The RSNs are ranked by medians. (*D*) A bar plot depicting correlations between the observed and predicted factor scores of different facets of cognitive ability, using ROIs from all networks and each separate RSN as predictors. The RSNs were ranked according to the correlations between the observed and predicted *g* scores (left to right). (*E*) The same as (*D*) but using entropy profile after deleting the unreliable ROIs. ^*^ and ^**^ above the bars denote the corresponding significance levels of *P* < 0.05 and *P* < 0.01, respectively, after applying the correction of FDR < 0.05. The error bars indicate the 95% CI.

The reliability guaranteed the upper bound of functional validity of individual entropy profile. Next, we will assess the validity of entropy profile through the prediction of cognitive abilities. We obtained two-section-averaged individual entropy profiles with deletion of unreliable ROIs and applied these profiles in an attempt to predict individual cognitive abilities. First, we adopted a confirmatory factor analysis (CFA) model based on several behavioral tasks (Dubois et al. 2018b) to estimate the cognitive ability scores of each individual, as described in the Materials and Methods. Figure 4*A* displays the model structure along with the factor loading estimates. The depicted model yielded an acceptable fit of our data: ${\chi}_{(30)}^2$= 124.260, *P* = 0.000, CFI = 0.959, RMSEA = 0.054, and SRMR = 0.037. Note that the ${\chi}^2$-goodness of fit test is highly sensitive for large samples and the model fit is thus evaluated based on the alternative fit indices (CFI, RMSEA, and SRMR). Next, we used this model to compute the factor scores for individual participants. The estimated factor scores were perfectly correlated with the corresponding latent factors across participants (see Supplementary Fig. S4). We then performed predictions based on a multivariate regularized regression, using the individual entropy profiles as predictors and the cognitive ability factor scores as the outcome. Figure 4*B* illustrates the significant correlations of the predicted scores with the measured *g* scores across individuals (${r}_{(996)}$ = 0.319, *P* = 0.000, 95% CI = [0.265, 0.377]). These results suggest that an individual’s unique entropy profile can effectively reflect his/her cognitive ability. Similar to the identification results, we obtained a lower prediction effect size for the complexity measure when compared with previously reported predictions based on FC profiles (${r}_{(882)}$*=* 0.457, *P* = 0.000 as reported in Dubois et al. (2018b) and ${r}_{(996)}$*=* 0.443, *P* = 0.000, 95% CI = [0.396, 0.489] as reproduced in our dataset; see Supplementary Fig. S6*B*).

We further explored the distribution of the predictive power of each RSN. First, we observed natural variations in regression weights across iterations because of the LOFOV scheme. However, because the entropy in the ROIs was *z*-transformed before the regression analyses, the weights were comparable across the ROIs. An ROI with a consistently high weight across iterations was considered highly important. Therefore, we computed the mean absolute weight of each ROI across the LOFOV iterations. As illustrated in the violin plots in Figure 4*C* (and as a brain map in Supplementary Fig. S7*B*), the median distributions reflect the ROIs within the DMN, FP, and VIS networks that are highly likely to contribute to the prediction power.

Second, we applied ROIs corresponding to a given RSN to separate predictions of cognitive ability. The correlations between the predicted and observed *g* values in each subnetwork are provided in Figure 4*D*. The DMN and VIS networks exhibited strongest prediction powers, and the predictions were significant in every RSN except LIM, which may be caused of the removal of many ROIs with low reliability. The prediction process was then repeated for each specific cognitive ability factor (*vis*, *cry*, *mem*, and *spd* in Fig. 4*A*). As illustrated in Figure 4*D*, only *vis*, *cry*, and *mem* could be predicted successfully from the entropy profile when using ROIs belonging to all networks. The best predictive performance was achieved for *cry*. In addition, only some RSNs were predictive for specific cognitive ability scores, and the most predictive network differed with respect to different abilities. The ATT and DMN networks were ranked as the best predictors for *vis* and *mem*, respectively, whereas the SAL network was the best predictor for both *cry* and *spd*. The SAL and DMN networks appeared to yield significant predictive power for most of the specific cognitive abilities (SAL, 4 abilities at a *P* < 0.05 level; SAL and DMN, 3 abilities at a *P* < 0.01 level).

### Neuroanatomical Basis of the Individual Entropy Profile

Next, we performed a two-step exploration of the neuroanatomical (structural) basis of the individual entropy profile. First, we aimed to understand the apparent spatial heterogeneity in the entropy profile in reliable ROIs (17 ROIs with low test–retest reliability have been removed from the analysis). Here, we relied on the reliable entropy profile shared by the population, known as the entropy profile *blueprint*. The blueprint can also be considered a common coordinate for the comparison of groups and individuals. Second, we investigated which structural features determined individual differences in entropy profiles (i.e., fingerprints). A joint interpretation of the results of these 2 steps should facilitate a better understanding of individual entropy profiles as deviations from the blueprint. The unreliable ROIs were deleted to yield unbiased conclusions.

#### The Blueprint

**Figure 5:**
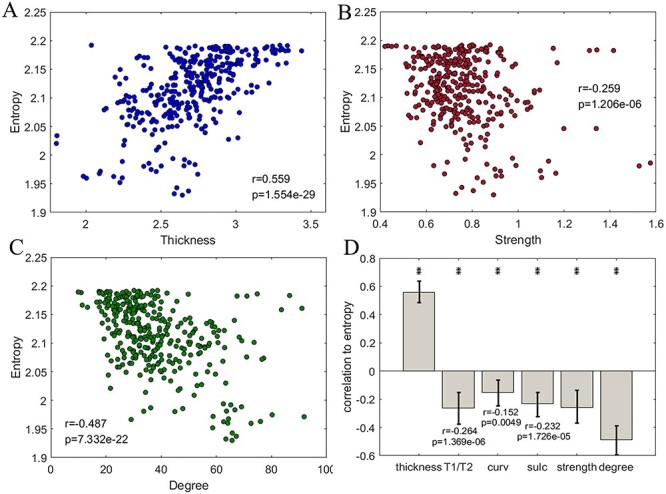
The cortical entropy profile as a blueprint and the corresponding structural foundation. (*A*) A scatter plot of the regional complexity versus the regional cortical thickness value (blueprint). (*B*) A scatter plot of the complexity blueprint versus the structural connectivity strength blueprint. The structural network data are displayed at a connectivity threshold of ${w}_{ij}$ = 0.001. The same threshold was applied to the following subfigures. (*C*) A scatter plot of the complexity blueprint versus the structural connectivity degree blueprint. (*D*) A bar plot of the correlations between the complexity blueprint and structural property blueprints. ^*^ and ^**^ indicate *P* < 0.05 and *P* < 0.01, respectively, after correction at an FDR of < 0.05. The error bars indicate the upper and lower bound of 95% CI.

After obtaining the reliable entropy profile blueprint, we associated the entropy profile blueprint with the blueprints of multiple structural properties, including cortical thickness, myelin content (T1/T2), cortical curvature (curv), sulci depth (sulc), connectivity strength, and degree. Notably, the regional cortical thickness was positively associated with the entropy across cortical regions (Fig. 5*A*, ${r}_{(358)}$ = 0.559, *P* = 0.000, 95% CI = [0.480, 0.631]), whereas entropy was negatively associated with the structural connectivity strength (Fig. 5*B*, ${r}_{(358)}$ = −0.259, *P* = 0.000, 95% CI = [−0.376, −0.153]). This latter association strengthened further when degree measure was used (Fig. 5*C*, ${r}_{(358)}$ = −0.487, *P* = 0.000, 95% CI = [−0.587, −0.382]). A summary of the group-level entropy-structure correlations in a bar plot depicted in Figure 5*D* demonstrates that all associations were significant at the *P* < 0.05 level, even after correcting for multiple testing. However, only the association with cortical thickness was positive, and all other associations were negative. The results in Figure 5*C*,*D* were derived from structural networks after thresholding at ${w}_{ij}$ = 0.001, as described for other analyses. Note that quantitatively similar results were derived using other threshold values (see Supplementary Fig. S1*B*).

#### The Fingerprint

**Figure 6:**
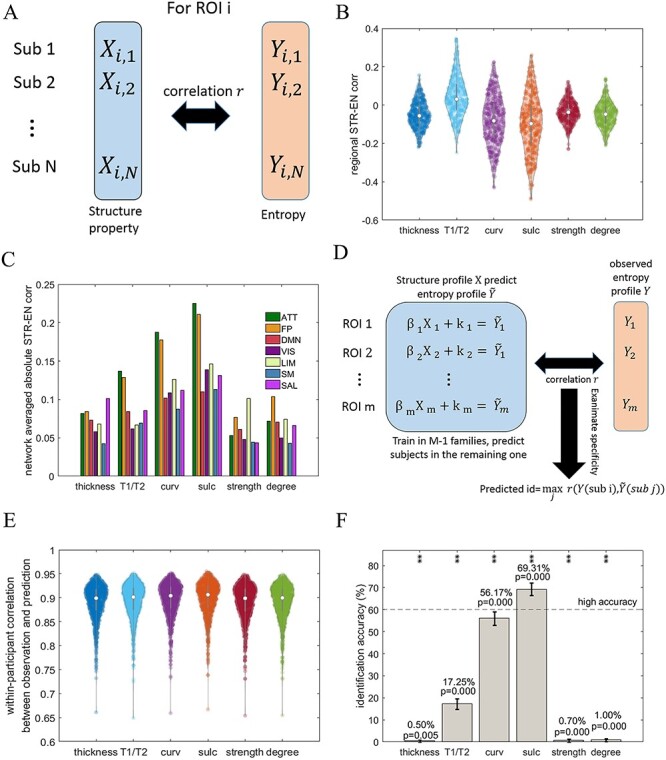
The structural basis of the individual entropy profile. (*A*) A schematic overview of the computation approach used to estimate the association between the brain structure and complexity across individuals in each ROI. (*B*) The distributions of regional structure–entropy associations across individuals with respect to different structural properties. The network connectivity (strength and degree) results are illustrated using a connectivity threshold of ${w}_{ij}$= 0.001. The same threshold was applied in the following panels. (*C*) A bar plot of the network-averaged regional structure–entropy correlations derived using different structural properties. (*D*) The model used to predict cortical entropy profiles using individual structural properties, together with the examination of specificity across individuals. (*E*) Violin plots depicting the correlations between the observed and predicted entropy profiles for the same participant according to different structural properties. (*F*) The accuracies of identification based on observed and predicted individual cortical entropy profiles when using different structural properties. ^*^ and ^**^ above the bars denote the corresponding significance levels of *P* < 0.05 and *P* < 0.01 obtained by the permutation test, respectively, after applying the correction of FDR < 0.05. The error bars indicate the 95% CI.

Next, we investigated whether interparticipant variations in the entropy profiles were determined by individual differences in brain structural profiles. Here, we considered the same set of structural properties used in the blueprint analysis, as all the properties were proven previously to vary across individuals (Rauch et al. 2005; Wright et al. 2006; Holmes et al. 2012; Bjørnebekk et al. 2013; Riccelli et al. 2017; Toschi and Passamonti 2019; Liu et al. 2020). We first examined the cross-individual structure–entropy correlation in each ROI (Fig. 6*A*). These correlations differed widely across the ROIs (Fig. 6*B*). The estimated relationships associated with T1/T2, curv and sulc were distributed relatively broadly in the range of approximately −0.50 to 0.30. However, the distributions of correlations with cortical thickness, structural connectivity strength, and degree were relatively narrow, with ranges of −0.20 to 0.20. Next, we decomposed the global distributions into the 7 RSNs. The network-wise averaged values are displayed in Figure 6*C*. Among the considered structural features, T1/T2, curv and sulc exhibited the strongest correlations with entropy in the ATT, FP, and DMN networks. Importantly, our earlier analysis identified these networks as having the highest contribution in fingerprint identification based on reliable entropy profiles (Fig. 3*G*,*H*), which can be roughly interpreted as these RSNs have most degree of individual difference. This observation suggests that the individual entropy profiles are more strongly associated with individual differences in myelin, curv, and sulc profiles within the indicated subnetworks.

We then estimated a predictive model to further substantiate our findings (Fig. 6*D*). After training regression models for each ROI, the regional predictions were combined to generate predicted entropy profiles across ROIs. When we compared the similarity between the observed and predicted entropy profiles within the same participant, we observed that all structural properties predicted the profiles with similarly high levels of precision across the participants (Fig. 6*E*). Therefore, we further examined the cross-individual specificities of the predictions. We expected that the predicted structural brain property-based profile of 1 participant would be highly similar to the observed profile of the same participant but different from the observed profiles of other participants. Therefore, we subjected the observed and predicted entropy profiles to fingerprint identification and repeated this test for all structural properties (Fig. 6*F*). We only achieved the high level of identification accuracy (accuracy = 69.31%, *P* = 0.000, 95% CI = [66.55%, 72.02%]) with respect to the sulc-predicted entropy profile, suggesting that individual differences in sulc profiles largely explain the differences in observed entropy profiles between participants. The tests based on sulc, curv, and T1/T2 yielded relatively high levels of accuracy among all predications and were ranked similarly with respect to the network-wise mean correlations in the ATT and FP networks (Fig. 6*C*). Note that predictions based on the cortical thickness, structural connectivity strength, and degree yielded extremely poor accurate identifications. The results based on connectivity measures with other thresholds showed similarly low levels of accuracy (see Supplementary Fig. S1*C*). These results support the understanding of correlative analysis in Figure 6*C*, wherein changes in individual entropy profiles were influenced predominantly by sulc, curv, and myelin in individual brains.

## Discussion

In this work, we systematically investigated whether individual differences in cortical entropy profiles can be considered a stable trait and a predictor of cognitive ability. Additionally, we examined the specific anatomical features underlying spatial heterogeneity and individual differences in entropy profiles. Using established entropy profiles to identify specific individuals in a large dataset, we demonstrated the test–retest reliability and uniqueness of the individual whole cortical entropy profile. However, the test–retest reliability differs among the entropy profiles of 7 RSNs, highlighted by the LIM showing the lowest reliability. Furthermore, the predictive modelling framework demonstrated that the information from reliable cortical entropy profiles could effectively predict diverse facets of cognitive ability in individuals. We further determined that spatial variations in the entropy profile could be explained by the cortical thickness and structural connectivity, whereas interindividual variations between individual entropy profiles were determined by the sulc, curv, and myelin content. To the best of our knowledge, this study is the first to reveal the test–retest reliability, predictive power for cognitive ability, and neuroanatomical basis of the individual cortical entropy profile.

Our work addresses a crucial problem concerning the reproducibility and generalizability of results from entropy-based studies of individual differences. First of all, entropy is a measure of randomness and thus is sensitive to noise. The high noise level and low retest reliability shown in the central portions of the brain in the HCP preprocessed data is induced by limitations of fMRI technology, because of the far distance from the receiver coil (Glasser 2020). The noise-induced low retest reliability severely influenced the differentiation power of entropy profiles in the LIM network and impact on SAL networks, introducing a bias in the evaluation of individual differences among RSNs. In the present work, we aimed to achieve a balanced reliability among RSNs by a conservative removal of the low reliability ROIs from the analysis. However, a careful selection of the cutoff threshold, the balance among RSNs for the upper bound of the reliability, and ways to improve the signal-to-noise ratio to achieve more reliable signals in the LIM network and other affected networks should be investigated in future studies, in order to obtain a more convincing estimation of individual differences for these networks.

On the other hand, our findings validate the concept of the whole entropy profile based on rfMRI as a more stable marker and more informative predictor of the cognitive ability of an out-of-sample individual than profiles of any subsystems. A methodological framework to utilize the whole cortical entropy profile could ease the issue of imbalanced test–retest reliability showed in smaller scopes, i.e., RSN and ROI. In addition, whole cortical profile-based method offers advantageous in terms of integrating information when compared with previous correlation-based methods that considered spatial sites separately. In another area of research, previously proposed whole-brain analysis approaches used FC as a fingerprint and predictor of cognitive abilities (Finn et al. 2015; Dubois et al. 2018b). In a comparison of these FC-based methods with our entropy-based method, we observed some advantages of the former. Although entropy and FC profiles yielded comparable identification rates, the latter yielded a slightly more accurate prediction of cognitive performance. This slight superiority can be explained by 2 factors. First, it should be noted that the captured information is shared, to some extent, by the entropy- and FC-based characterizations of spatiotemporal brain activities. Several studies that analyzed fMRI data and optical voltage images have demonstrated a strong negative correlation between temporal signal complexity and FC strength (McDonough and Nashiro 2014; Wang et al. 2018; Liu et al. 2019). Second, FC profiles are advantageous because these can capture a much larger feature space ($O\big({N}^2\big)$) relative to that captured by entropy ($O(N)$). The increased features of FC approach could provide more reliable dimensions to compensate the noise-induced unreliability. Also a high-dimensional space can more sensitively distinguish between participants and predict their cognitive abilities. In this sense, the reliability and predictability of the entropy profile are likely to be limited by the exanimated feature dimension (i.e., number of ROIs or electrodes) and should be carefully checked when conducting a localized fMRI or EEG study.

In the relatively reliable part of cortical entropy profile, we revealed heterogeneity with respect to the degree of individual difference and predictive powers of different functional subsystems with identification and prediction of their cognitive abilities. Notably, the FP, ATT, and DMN networks were the greatest contributors to the reconducted identification of individuals. Previous studies have reported high levels of intertrial and interparticipant functional connectivity variability in these networks (Mueller et al. 2013). In our subsequent analysis, the FP and ATT networks were associated with the strongest structure-entropy associations, whereas the DMN appeared to have the highest predictive power for general cognitive ability. Overall, these findings demonstrate that the dynamic activities of these functional systems play a unique role in associations with the brain structure or cognitive ability. These observations are consistent with the “cortical hierarchy” hypothesis (Felleman and Van Essen 1991; Markov et al. 2014; Huntenburg et al. 2018), which proposed that higher-order systems (e.g., FP, ATT and DMN) control multimodal cognition and must be variable to support flexibility in functional processing. In contrast, lower-order systems (e.g., VIS and SM) are specialized to perform unimodal primary functions and require greater stability. However, we observed that the higher-order DMN network and lower-order VIS network were most predictive of general cognitive ability. Other subsystems were also identified as significant predictors of general cognitive ability. This observation suggests that general cognitive ability requires integration across the cortical hierarchy. This suggestion is plausible as the qualities of information processing in both the lower- and higher-order systems could be intuitively considered important for successful functional processing. Our findings thus provide further empirical support for the view that whole-brain approaches could better elucidate individual differences in general cognition. Additionally, the successful RSNs differed with respect to the predictions of different specific abilities. For example, the ATT network was a powerful predictor (evident by significance) of visuospatial and crystalized abilities but not of memory and processing speed. In addition, the DMN and SAL networks appeared to be the best predictors for memory and crystallized ability, respectively. Overall, our results suggest the involvement of different functional subsystems in the implementation of specific abilities, which is consistent with the perspective of functional specificity. Taken together, our observations reveal that information processing in the brain exceeds the constraints suggested by the hierarchical view. This revelation is consistent with the distributed information-processing theory (Colom et al. 2006; Gläscher et al. 2010) of general intelligence, as well as with flexible information routing when realizing specific abilities (Palmigiano et al. 2017; Wang and Yang 2018).

According to our results, the unique cortical entropy profiles of individuals could be attributable to interparticipant variations in cortical folding properties (sulc and curv) and myelination. In contrast, the individual cortical thickness and structural connectivity did not contribute to specific predictions. These findings regarding the anatomical determinants of entropy might improve our neurological understanding of brain pathologies and associated cognitive losses. Furthermore, the outcomes of this study can be used to more fully integrate the previous knowledge from several entropy-based and brain anatomy-based studies. For example, we might postulate that the previously observed loss of brain entropy in patients with Alzheimer’s disease (Mizuno et al. 2010; Yang et al. 2013; Azami et al. 2017; Wang et al. 2017) could be attributable to reductions in curv and sulc (Im et al. 2008; Liu et al. 2011, 2012).

In contrast to the above-described findings, the whole-brain entropy profile blueprint was based largely on spatial heterogeneity in cortical thickness and structural connectivity, consistent with recent studies that used spatial variation gradients of structural blueprints to explain inter-region differences in dynamics properties. For example, variations in neuronal oscillation time scales were linked to the spine densities of local neuronal circuits (Chaudhuri et al. 2015). Moreover, differences in outgoing regional FC patterns were associated with heterogeneity in myelination (Demirtaş et al. 2019). However, our study revealed discrepancies in the structure–dynamics relationship between the blueprint- and fingerprint-level analyses, as indicated by the functional consequences of differences in the brain structure between brain regions versus those across participants. For example, inter-regional changes in structural connectivity (e.g., strength and degree) were consistently and negatively coupled with complexity in the functional resting-state complexity. However, the interparticipant differences in connectivity formed both positive and negative associations with different regions. The observed discrepancies in the correlation patterns between blueprint and fingerprint analyses could be explained from the perspective of the complex systems theory. Entropy across the spatial region is the result of network integration based on different anatomical and connectivity measures. Consequently, there may be significant correlations between each measure, although some measures may not have a causal effect on the measured dynamical complexity (e.g., thickness, despite the lack of effect of this measure on the individual fingerprint). Our findings clearly demonstrate that blueprint-based neuroscience studies do not fully elucidate the structure–dynamics relationship and that the determination of additional information at the individual level is warranted. Our analyses also suggest that future studies should implement more complex structure–dynamics interactions in the high-dimensional system and consequently derive a unified principle that can explain the observations from both blueprint- and fingerprint-based studies.

## Notes

Data were provided by the Human Connectome Project, WU-Minn Consortium (Principal Investigators: David Van Essen and Kamil Ugurbil; 1U54MH091657) funded by the 16 National Institute of Health (NIH) Institutes and Centers that support the NIH Blueprint for Neuroscience Research; and by the McDonnell Center for Systems Neuroscience at Washington University. *Conflict of Interest*: None declared.

## Funding

Hong Kong Baptist University (HKBU) Research Committee Interdisciplinary Research Matching Scheme 2018/19 (IRMS/18-19/SCI01); Germany-Hong Kong Joint Research Scheme (G_HKBU201/17 awarded to CSZ and ID 57391438 awarded to AH). This research was conducted using the resources of the High-Performance Computing Cluster Centre at HKBU, which receives funding from the Hong Kong Research Grant Council and HKBU.

## Supplementary Material

FIGS1_tgaa015

FIGS2_tgaa015

FIGS3_tgaa015

FIGS4_tgaa015

FIGS5_tgaa015

FIGS6_tgaa015

FIGS7_tgaa015

Supplementary_Material_tgaa015
