# Supplementary figures and images for: Individual Cortical Entropy Profile: Test–Retest Reliability, Predictive Power for Cognitive Ability, and Neuroanatomical Foundation
Source: Cereb Cortex Commun. 2020 May 7;1(1):tgaa015. doi: 10.1093/texcom/tgaa015 (PMC8153045; doi:10.1093/texcom/tgaa015)

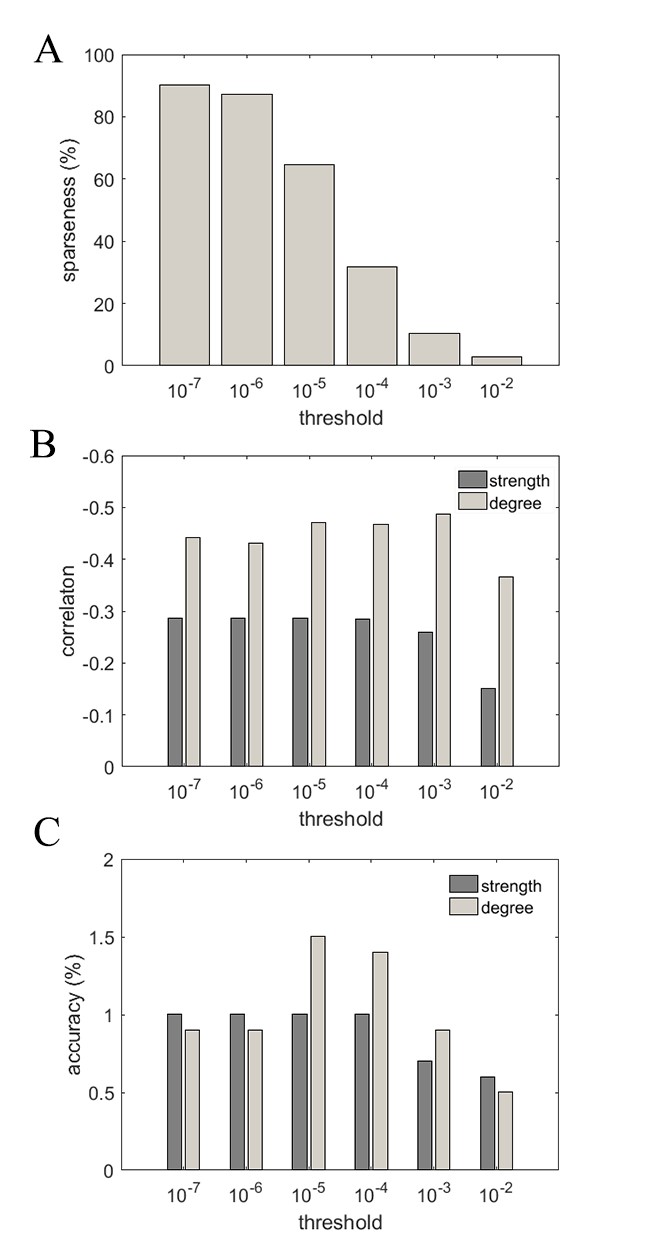

Supplement: FIGS1_tgaa015 [file figs1_tgaa015.jpeg]

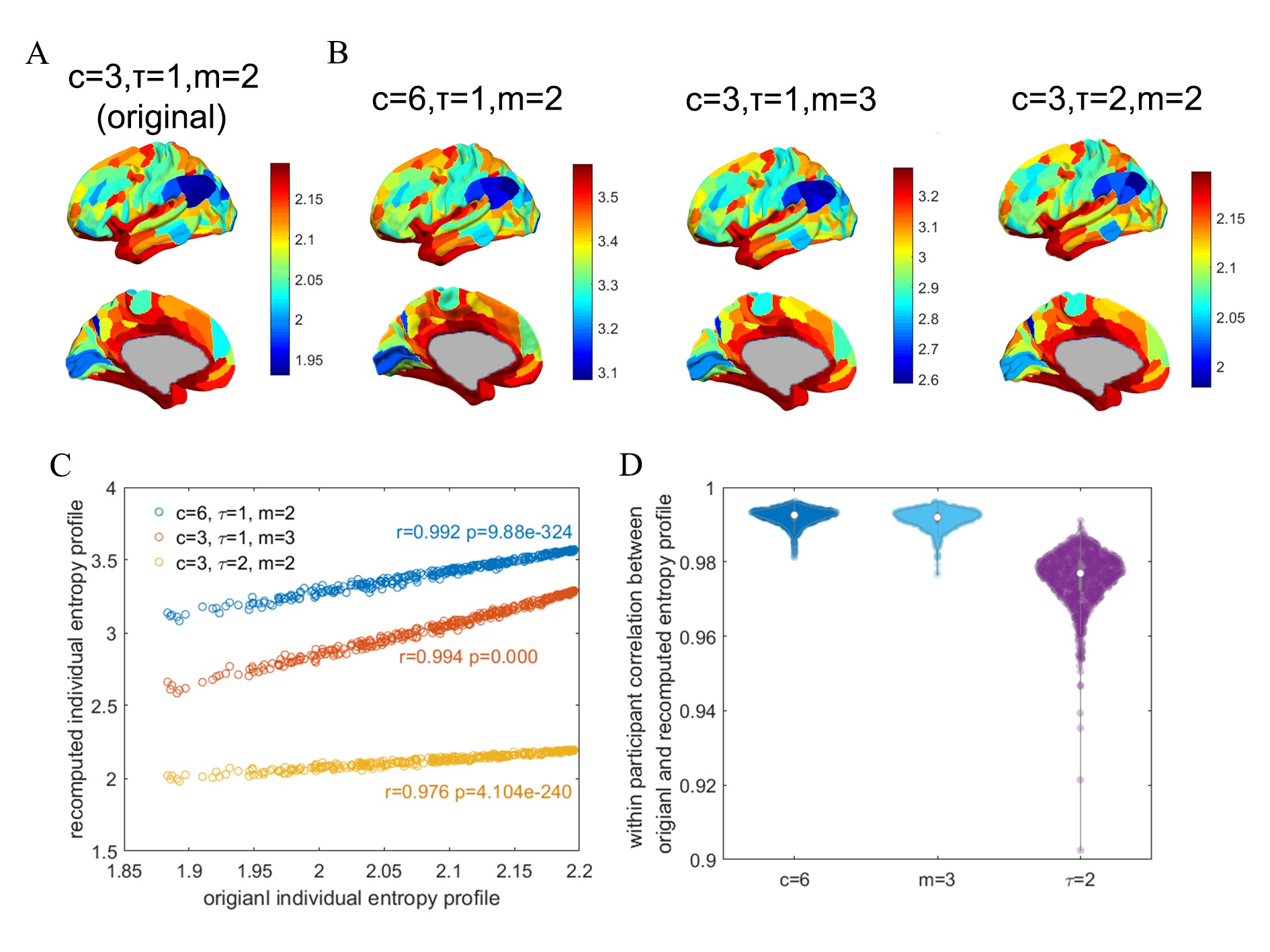

Supplement: FIGS2_tgaa015 [file figs2_tgaa015.jpeg]

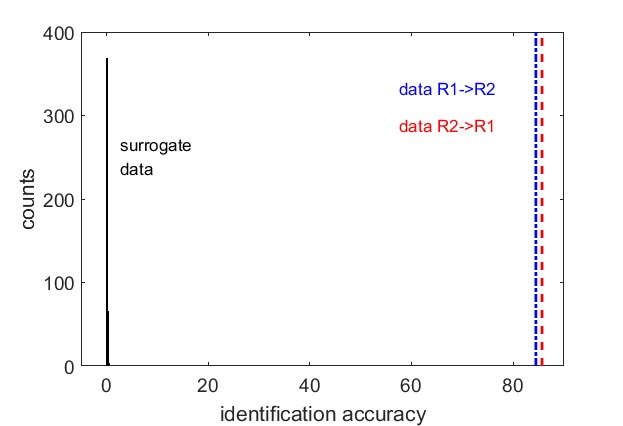

Supplement: FIGS3_tgaa015 [file figs3_tgaa015.jpeg]

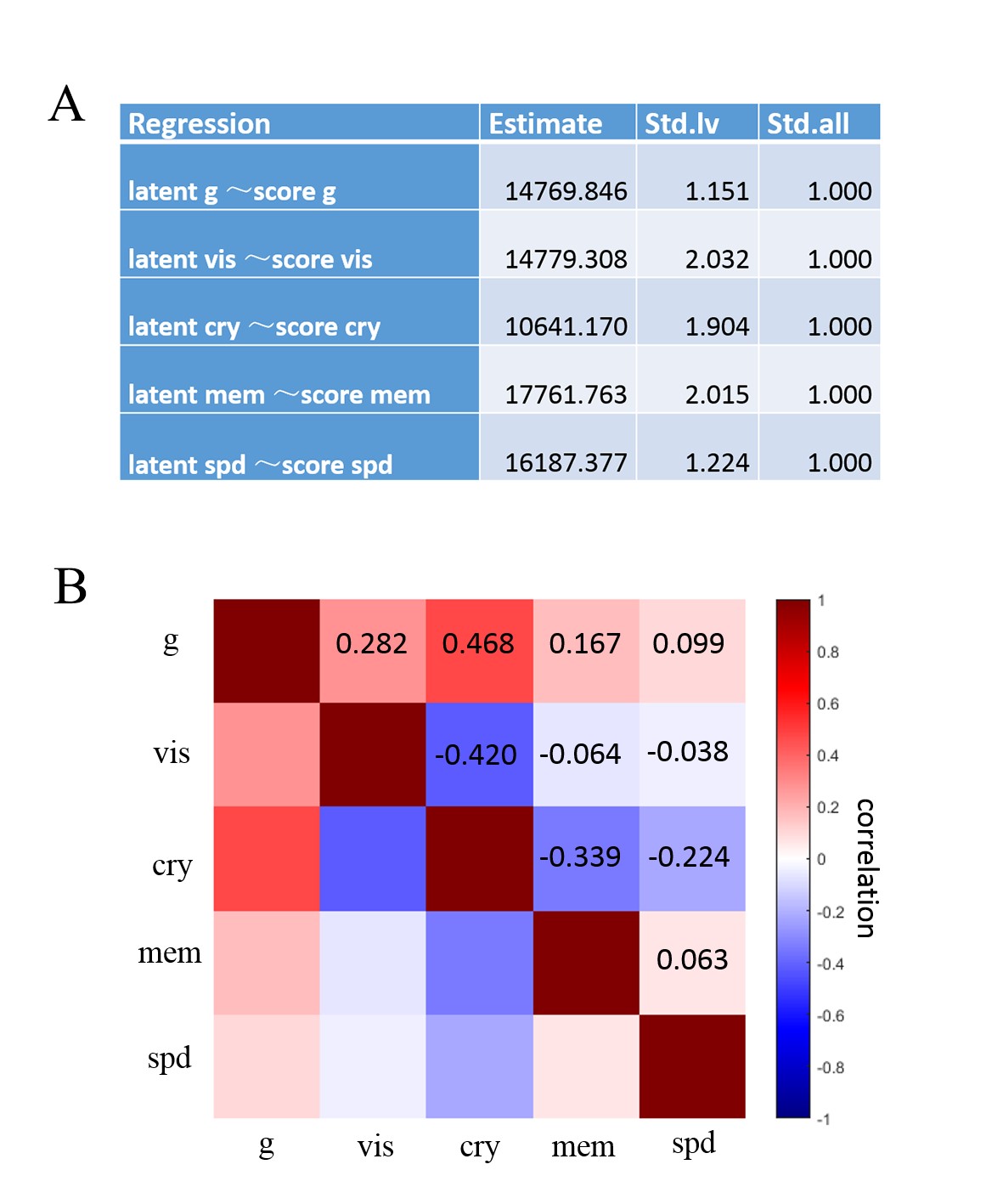

Supplement: FIGS4_tgaa015 [file figs4_tgaa015.jpeg]

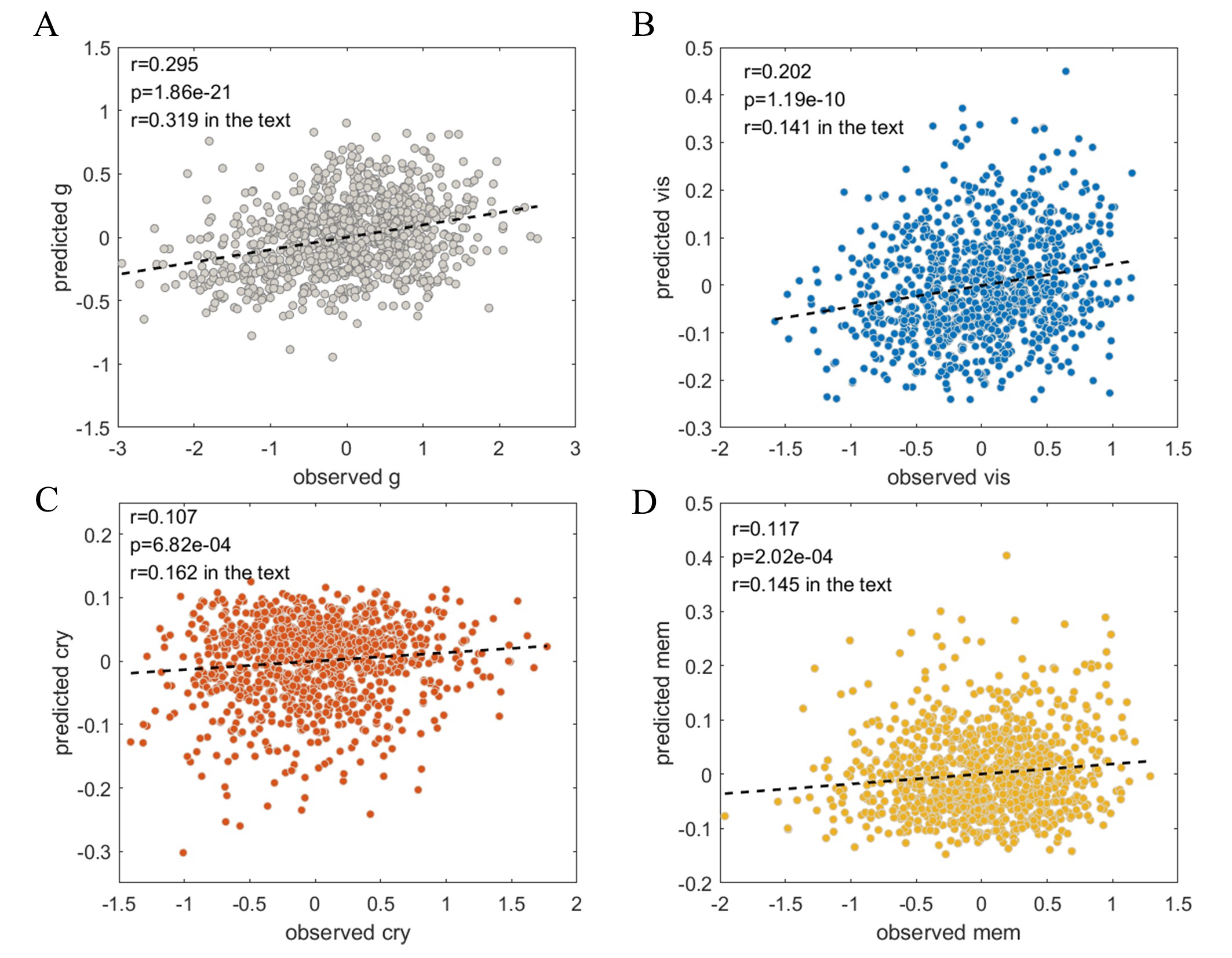

Supplement: FIGS5_tgaa015 [file figs5_tgaa015.jpeg]

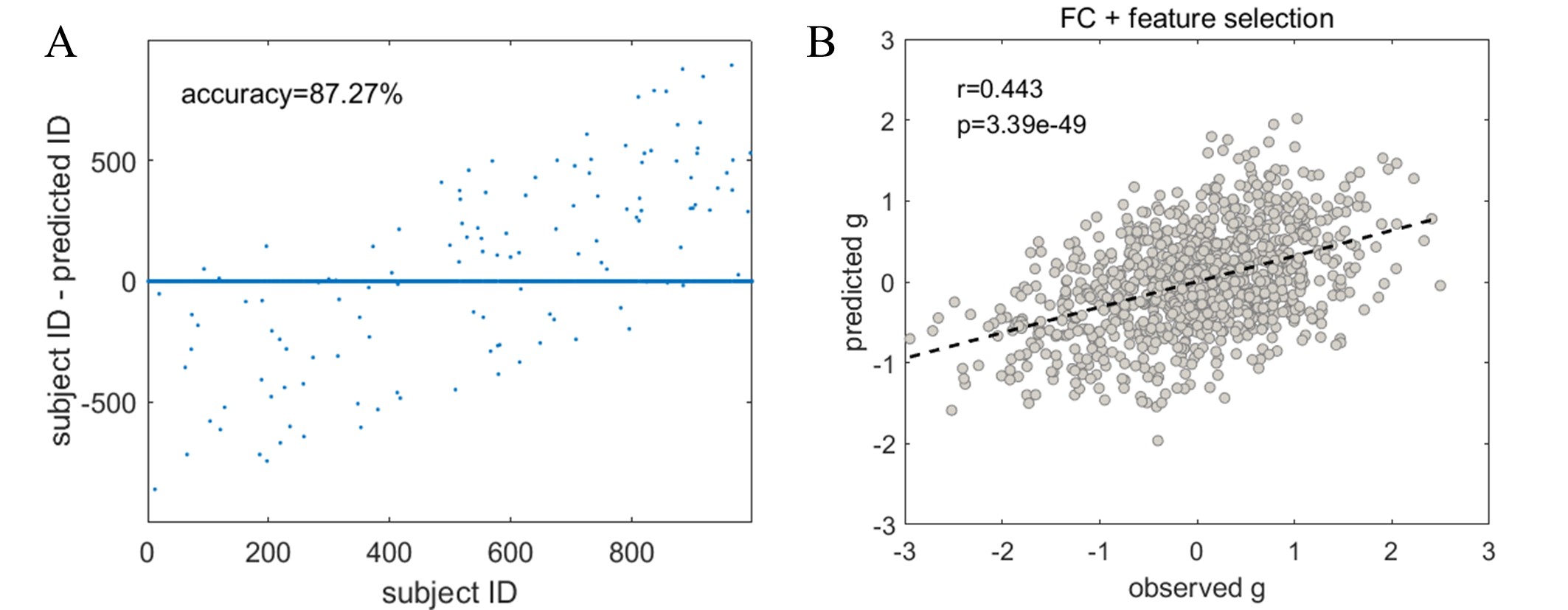

Supplement: FIGS6_tgaa015 [file figs6_tgaa015.jpeg]

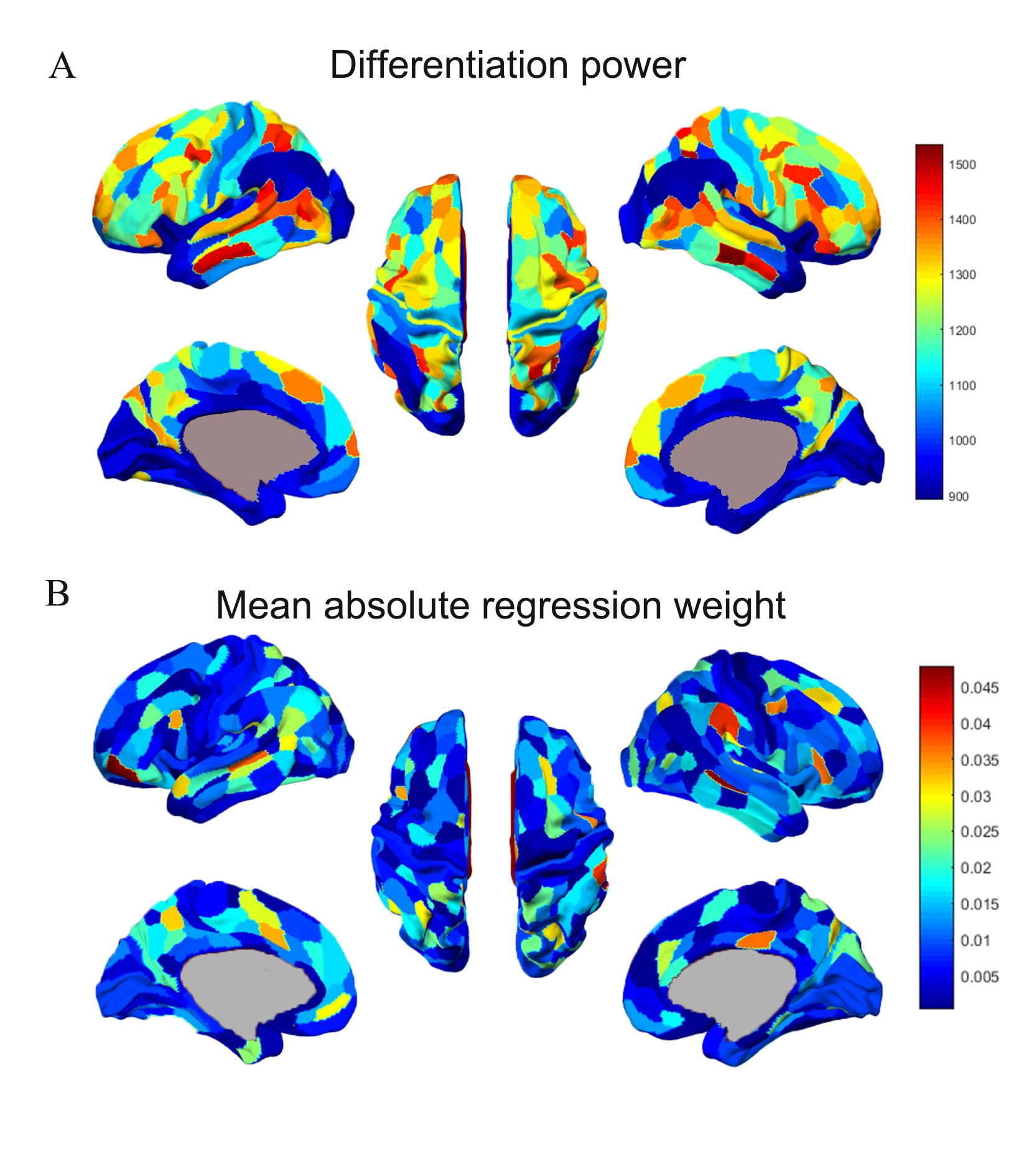

Supplement: FIGS7_tgaa015 [file figs7_tgaa015.jpeg]
