# Supplementary material for: Individual Cortical Entropy Profile: Test–Retest Reliability, Predictive Power for Cognitive Ability, and Neuroanatomical Foundation
Source: Cereb Cortex Commun. 2020 May 7;1(1):tgaa015. doi: 10.1093/texcom/tgaa015 (PMC8153045; doi:10.1093/texcom/tgaa015)
Supplement: Supplementary_Material_tgaa015 [file supplementary_material_tgaa015.docx]

**Supplementary Material**


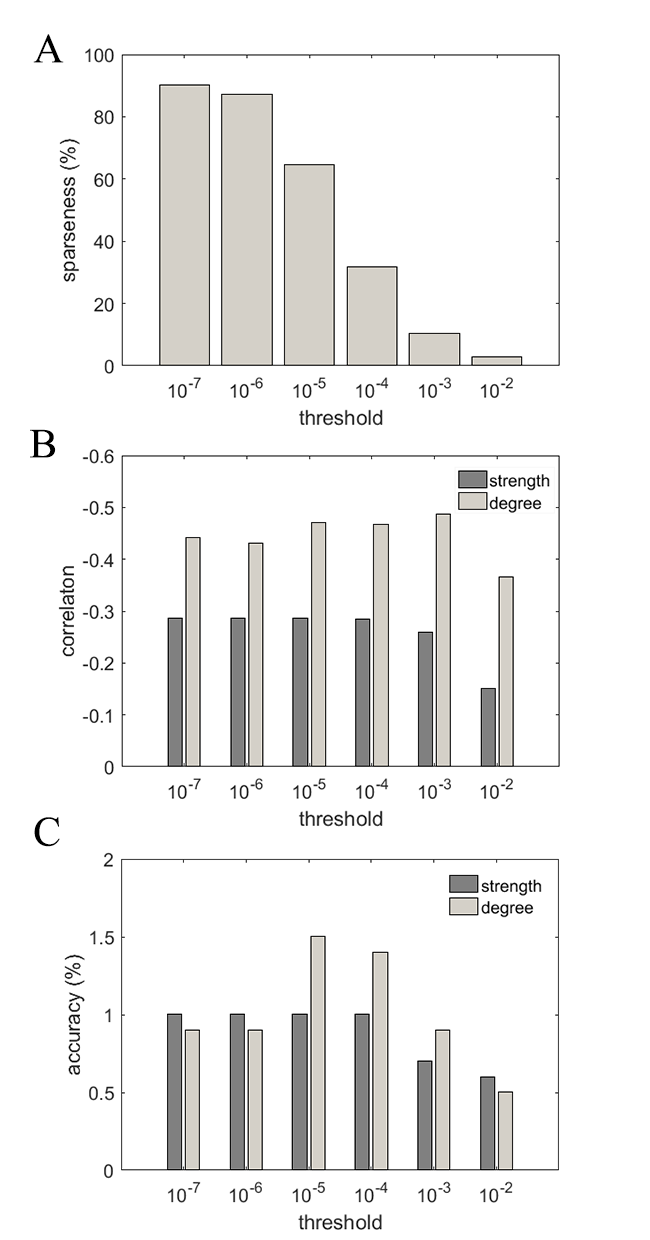


Figure S1. Effects of thresholding in DTI. (A) Corresponding to the Materials and Methods section. The average sparseness (percentage of existing links) of individual structural network under different thresholds. (B) Corresponding to Fig. 5D. The correlation between entropy and strength/degree in blueprint analysis under different thresholds. (C) Corresponding to Fig. 6F. The accuracy of individual entropy profile identification based on strength/degree under different thresholds. Notably, connectivity degree exhibited greater power than strength in explaining the variation of entropy blueprint and fingerprint.


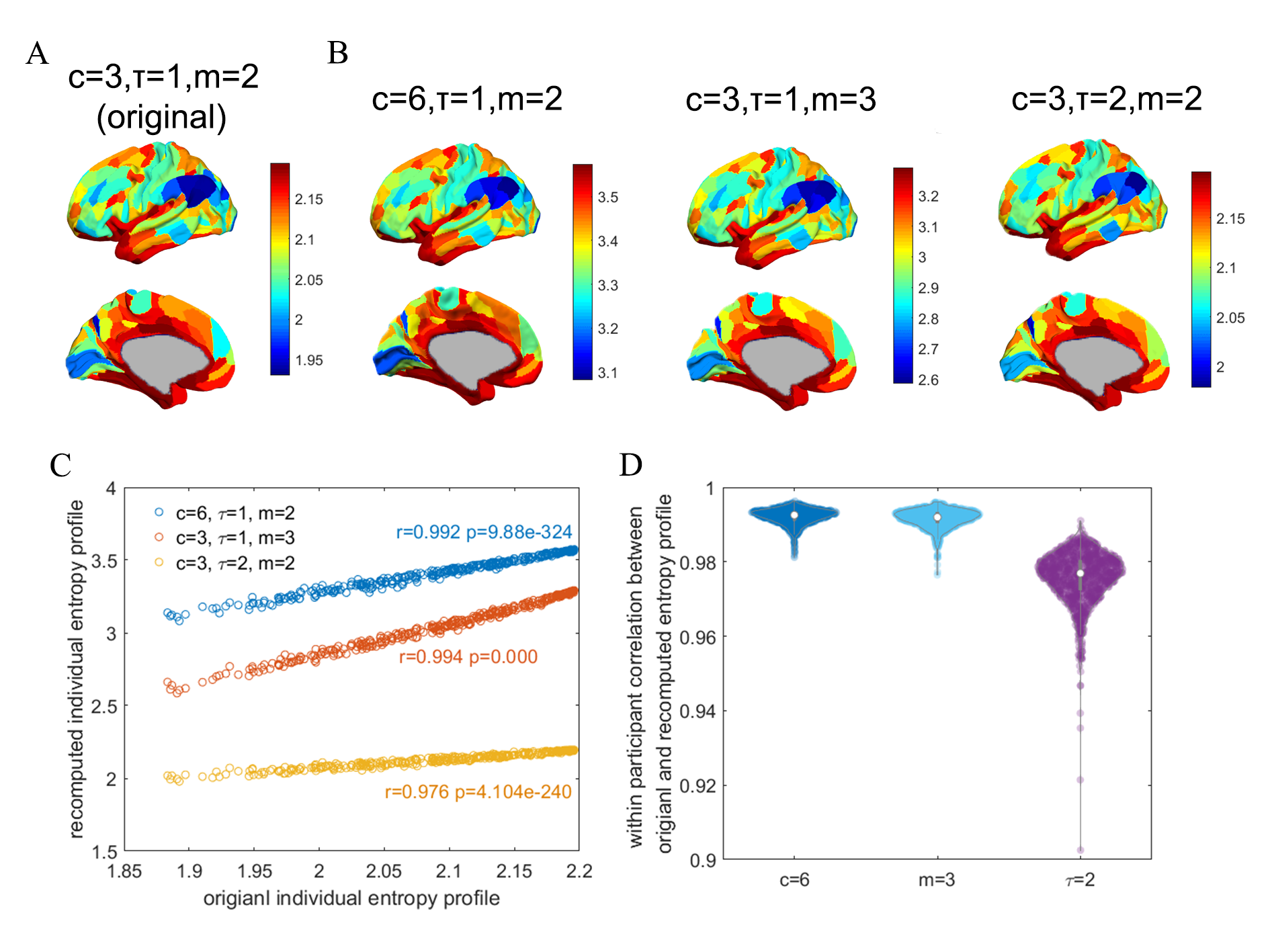


Figure S2. The effect of parameters on estimating individual entropy profile. The data of run 1 of REST1 was used for illustration. (A) The brain map of entropy profile in exemplified subject using the original parameter setting, i.e. c = 3, τ = 1 and m = 2. (B) The brain map of entropy profile in exemplified subject using changed parameters c, m or τ. (C) Scatter plot using data in (B) against data in (A). The entropy profiles from changed parameter settings are highly correlated with the original profile. (D) Violin lot for within-participant correlation between original entropy profile and recomputed entropy profile. The results suggested only increase of τ could slightly influence the estimation of entropy profile. This is in line with the understanding that increasing c and m will increase the resolution of temporal pattern searching, while increase τ will reduce the resolution.

**
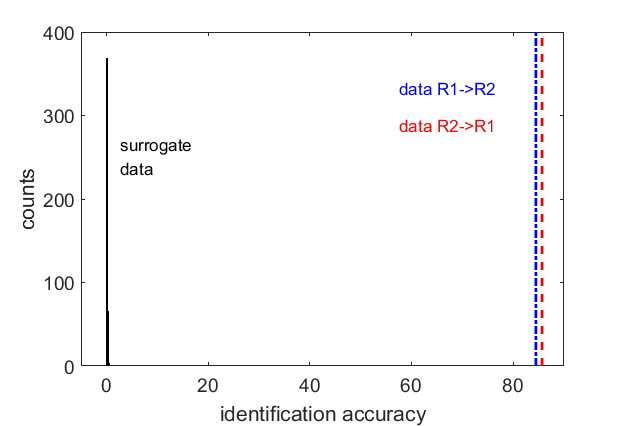
**

Figure S3. Example of generating surrogate data to assess significance of identification based on whole cortex entropy profile. Corresponding to the Materials and Methods section and Fig. 2C. The black bars illustrate the distribution of generated surrogate data in 1000 times realization according to the permutation test outlined in the Materials and Methods section. The blue dash dotted line and red dashed line indicate the obtained accuracy in real data, which lies far away from the distribution of surrogated data. Similarly, in other permutation tests, the surrogated data were concentrated near zero and the achieved maximum accuracy was 0.60%.


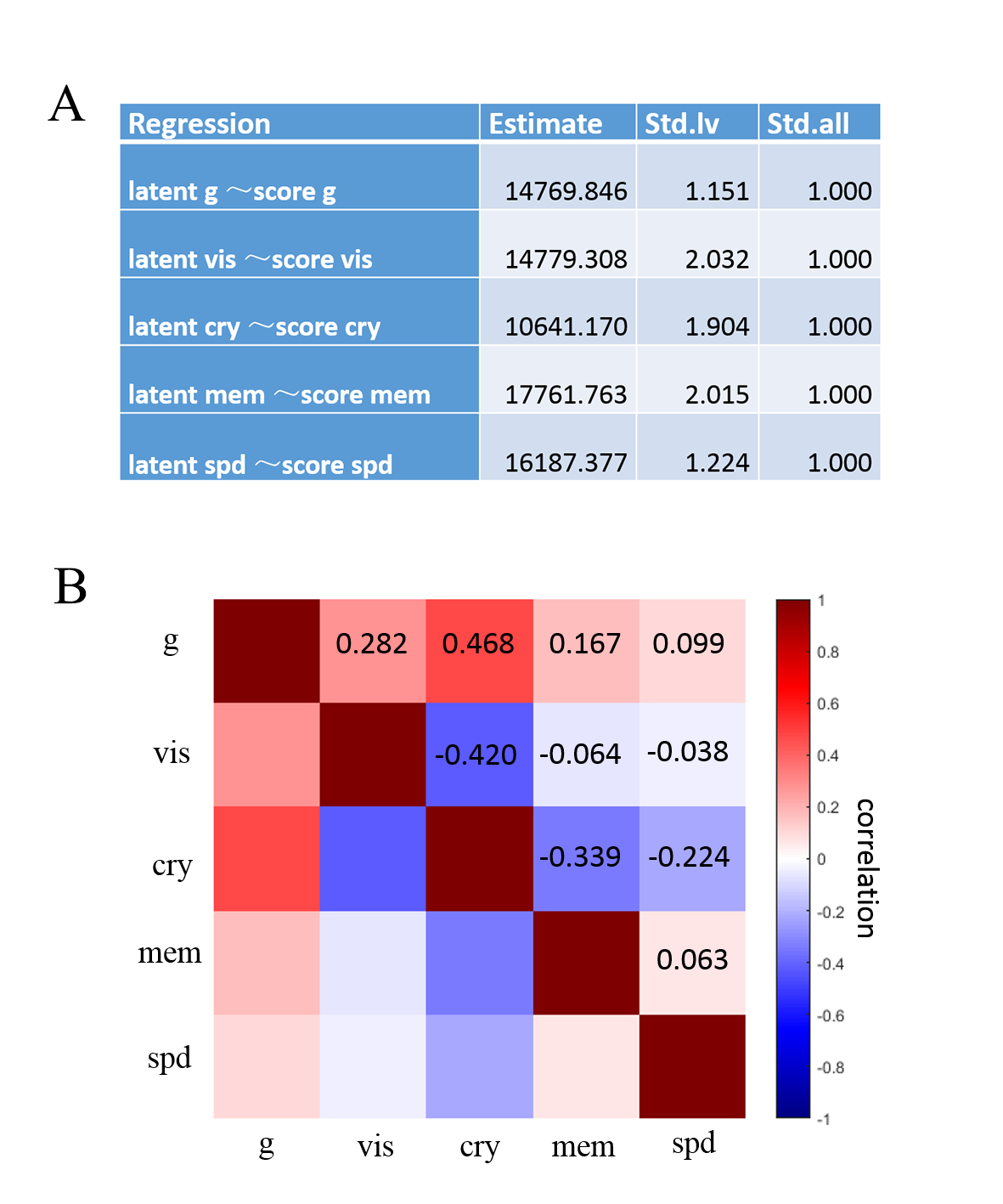


Figure S4. Evaluating factor score estimations. Corresponding to the Materials and Methods section. (A) Regression coefficients between latent variables and computed factor scores. The column “Std.all” indicates inter-participant correlations. (B) Correlations between factor scores generated from CFA model, which are supposed to be all zero under the assumptions of the estimated CFA.


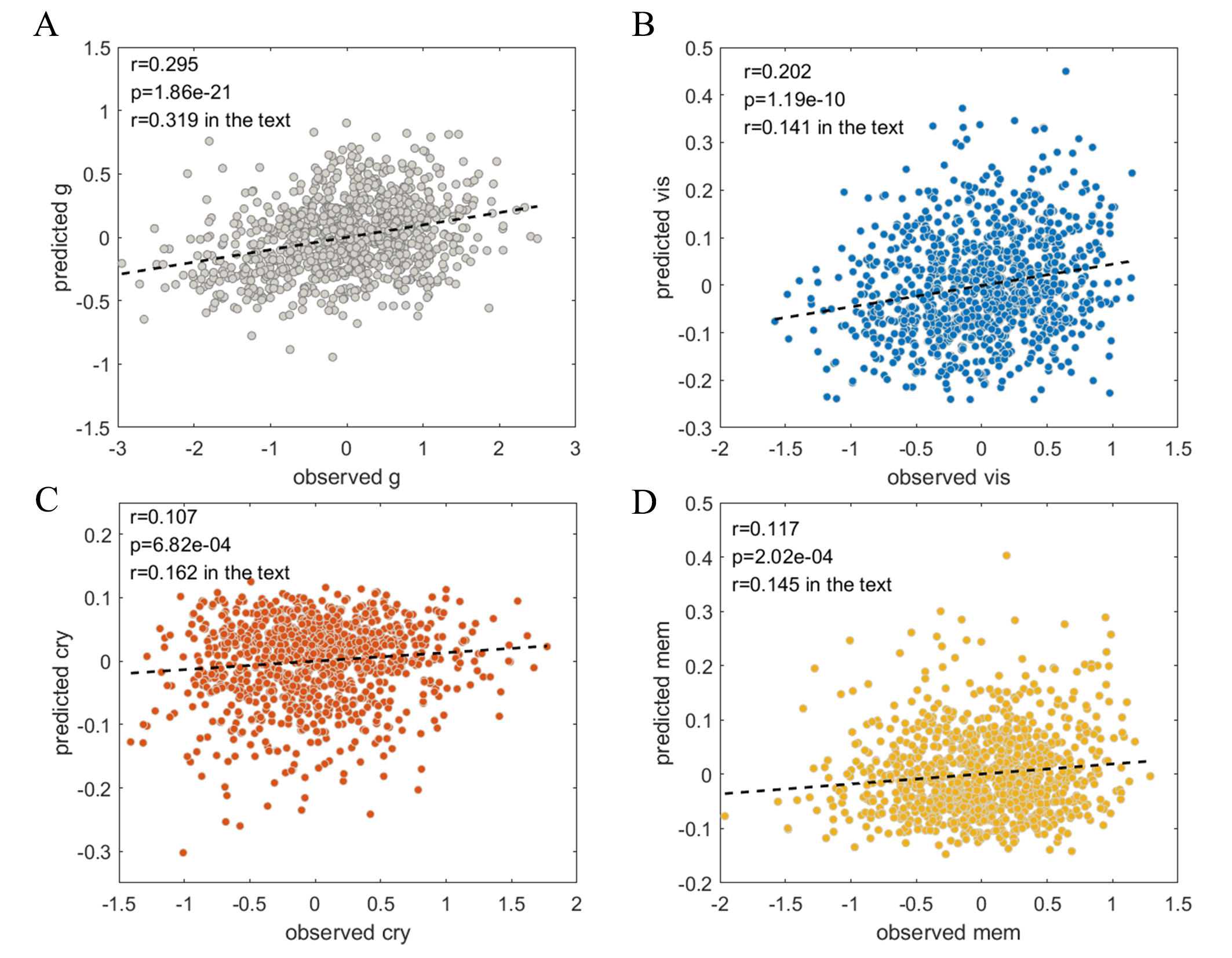


Figure S5. Cognitive ability prediction using the algorithm proposed in previous literature. In previous studies using FC profiles (Finn et al. 2015; Dubois et al. 2018b), before training the multivariate regression model, the correlations between each predictor and the ability score were first examined to reduce the feature space. The predictors showing no significant correlation under a given *p*-value threshold $p_{th}$ were excluded. However, in our entropy profile-based analysis, we found this manual selection of features to decrease the final prediction performance. The results are given: (A) Corresponds to Fig. 4B. Scatter plot of observed *g* vs. predicted *g* based on entropy profile in the whole cortex under $p_{th}$< 0.01; (B) Corresponds to Fig. 4D. Scatter plot of observed *vis* vs. predicted *vis* based on entropy profile in the whole cortex under $p_{th}$< 0.01. (C) Corresponds to Fig. 4D. Scatter plot of observed *cry* vs. predicted *cry* based on entropy profile in the whole cortex under $p_{th}$< 0.01. (D) Corresponds to Fig. 4D. Scatter plot of observed *mem* vs. predicted *mem* based on entropy profile in the whole cortex under $p_{th}$< 0.01. The corresponding results in the text (without *p*-value thresholding) are indicated in each subplot.


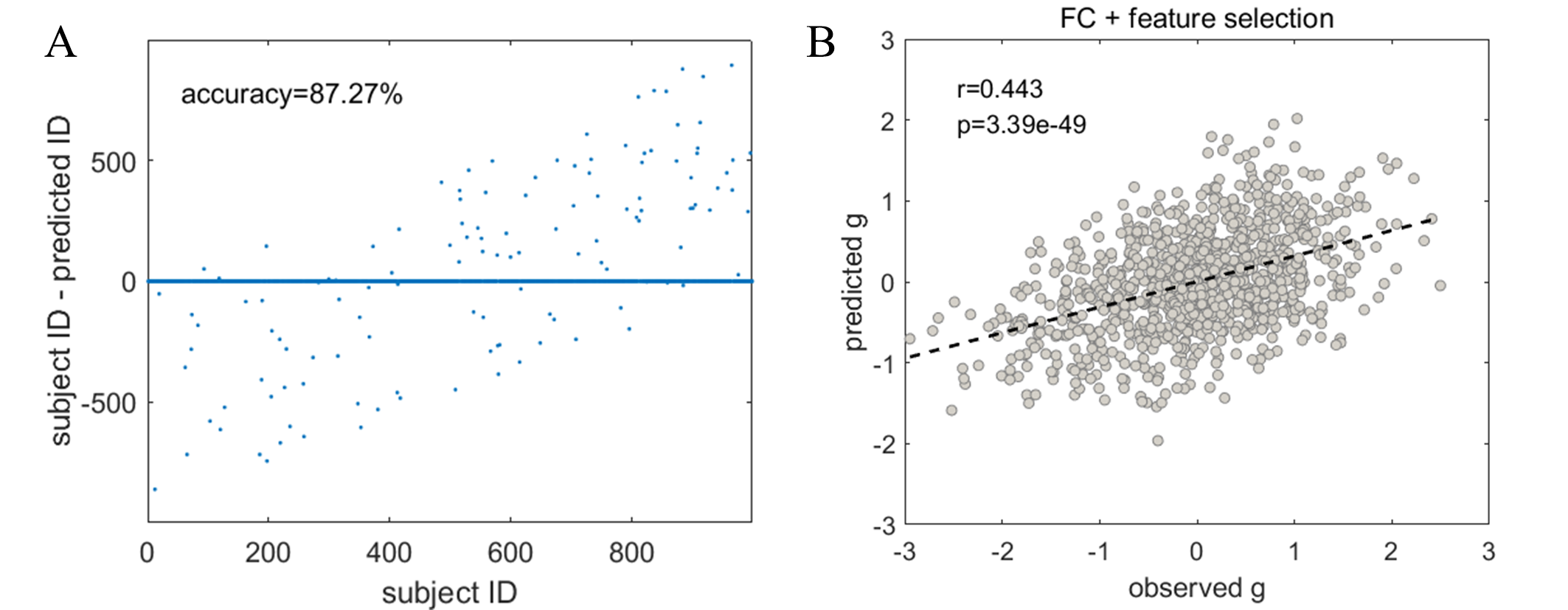


Figure S6. Identification and cognitive ability prediction based on functional connectivity (FC) profile. For comparison, the original FC-profile-based identification and cognitive ability prediction (Finn et al. 2015; Dubois et al. 2018b) were implemented. The individual FC matrix (360$\times$360) from two different days were vectorized (upper triangle elements, 1$\times$ 64620) and input to the calculation of correlation and regression model. The remaining procedures were as described in the Materials and Methods section. (A) Corresponds to Fig. 2C. Scatter plot illustrating the accuracy of predicted participant ID using REST1 to predict REST2 based on FC profiles. (B) Corresponds to Fig. 4B. Scatter plot of observed *g* vs. predicted *g* based on FC profile in the whole cortex. The unreliable ROI was not removed. The displayed results are from the best setting of the ridge regression penalty $\lambda$. Note that we conducted the feature selection step ($p_{th}$< 0.01) as described in the Materials and Methods section, for the computational cost to perform pure ridge regression (about 30 hours to search one $\lambda$).


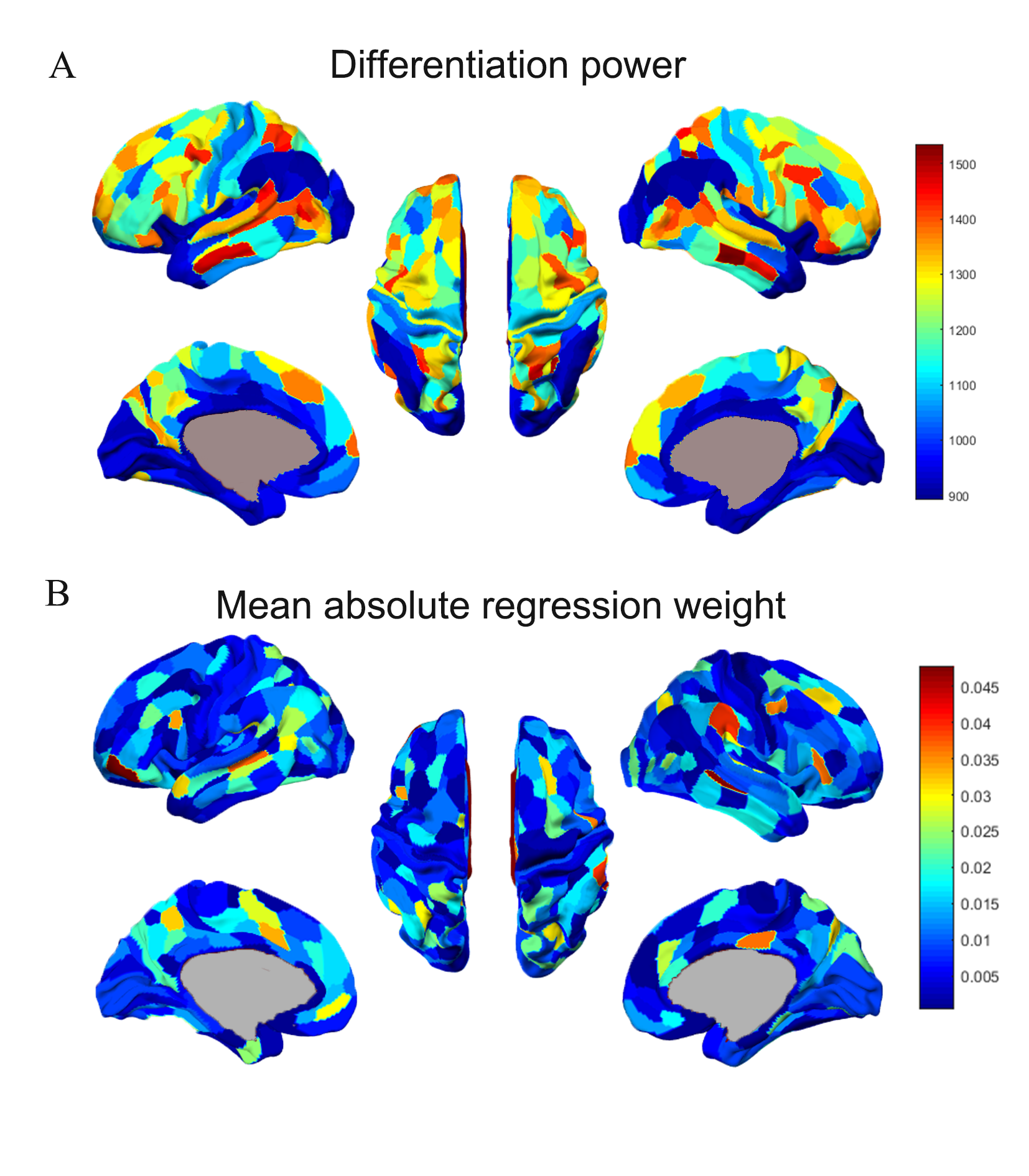


Figure S7. (A) Spatial distribution of differentiation power and (B) mean absolute regression weight during LOFOV for general ability *g*. They correspond to Fig. 2E and Fig. 4C, respectively.
